# Functional Characteristics of the Flying Squirrel's Cecal Microbiota under a Leaf-Based Diet, Based on Multiple Meta-Omic Profiling

**DOI:** 10.3389/fmicb.2017.02622

**Published:** 2018-01-04

**Authors:** Hsiao-Pei Lu, Po-Yu Liu, Yu-bin Wang, Ji-Fan Hsieh, Han-Chen Ho, Shiao-Wei Huang, Chung-Yen Lin, Chih-hao Hsieh, Hon-Tsen Yu

**Affiliations:** ^1^Department of Life Science, National Taiwan University, Taipei, Taiwan; ^2^Genome and Systems Biology Degree Program, National Taiwan University & Academia Sinica, Taipei, Taiwan; ^3^Institute of Information Science, Academia Sinica, Taipei, Taiwan; ^4^Department of Anatomy, Tzu Chi University, Hualien, Taiwan; ^5^Institute of Oceanography, National Taiwan University, Taipei, Taiwan; ^6^Institute of Ecology and Evolutionary Biology, National Taiwan University, Taipei, Taiwan; ^7^National Center for Theoretical Sciences, Taipei, Taiwan

**Keywords:** animal-microbe interaction, ecological adaptation, gut microbiota, metabolomics, metagenomics, metatranscriptomics

## Abstract

Mammalian herbivores rely on microbial activities in an expanded gut chamber to convert plant biomass into absorbable nutrients. Distinct from ruminants, small herbivores typically have a simple stomach but an enlarged cecum to harbor symbiotic microbes; however, knowledge of this specialized gut structure and characteristics of its microbial contents is limited. Here, we used leaf-eating flying squirrels as a model to explore functional characteristics of the cecal microbiota adapted to a high-fiber, toxin-rich diet. Specifically, environmental conditions across gut regions were evaluated by measuring mass, pH, feed particle size, and metabolomes. Then, parallel metagenomes and metatranscriptomes were used to detect microbial functions corresponding to the cecal environment. Based on metabolomic profiles, >600 phytochemical compounds were detected, although many were present only in the foregut and probably degraded or transformed by gut microbes in the hindgut. Based on metagenomic (DNA) and metatranscriptomic (RNA) profiles, taxonomic compositions of the cecal microbiota were dominated by bacteria of the Firmicutes taxa; they contained major gene functions related to degradation and fermentation of leaf-derived compounds. Based on functional compositions, genes related to multidrug exporters were rich in microbial genomes, whereas genes involved in nutrient importers were rich in microbial transcriptomes. In addition, genes encoding chemotaxis-associated components and glycoside hydrolases specific for plant beta-glycosidic linkages were abundant in both DNA and RNA. This exploratory study provides findings which may help to form molecular-based hypotheses regarding functional contributions of symbiotic gut microbiota in small herbivores with folivorous dietary habits.

## Introduction

Mammals and their gut microbiota have co-evolved for millions of years, forming an interdependent, symbiotic relationship (Stevens and Hume, 1998; Ley et al., 2008; Leser and Molbak, 2009). Establishing a cooperative association is particularly crucial for mammalian herbivores, as they heavily rely on gut microbiota to convert plant biomass into absorbable nutrients (Wallace, 1992; Kamra, 2005; Deusch et al., 2017). To provide space for gut microbiota, mammalian herbivores typically have a complex digestive tract with an enlarged compartment (Hume, 1989). For example, ruminants (so-called “foregut fermenters”), have specialized stomach chambers to house microbes for hydrolyzing and fermenting plant fibers. In contrast, other herbivores have an enlarged chamber in the large intestine (“hindgut fermenters”) to store ingesta for microbial activities (Mackie, 2002). Specifically, small mammalian herbivores usually have a well-developed cecum with a capacity ~10 times that of their stomach (Manning et al., 1994; Campbell et al., 2000). More interestingly, their gut structure apparently has a special sorting mechanism at the ileal-cecal-colic junction, permitting fluid and fine particles (including microbes and fine plant debris) to be retained in the cecum, while concurrently allowing coarse dry matter to rapidly pass through the gut (Hume, 2002). Such digestive strategies are believed to satisfy energy demands of small mammalian herbivores with high mass-specific metabolic rates (Sakaguchi, 2003). However, in contrast to numerous studies on ruminants and ruminal microbiota (Brulc et al., 2009; Weimer, 2015; Mao et al., 2016; Deusch et al., 2017), much less is known about co-adaptation characteristics between small mammalian herbivores and their cecal microbiota.

In this study, the white-faced flying squirrel (*Petaurista alborufus lena*) inhabiting montane areas of Taiwan (Oshida et al., 2011) was selected as a target organism. We focused on a wild species, instead of domestic animals, as wild herbivores usually have a great challenge to gain sufficient energy from coarse plant material. Consequently, studies on their gut microbiota were expected to advance knowledge regarding microbial functional characteristics under specific host dietary preferences in natural habitats (Hird, 2017). Compared to other palatable plant-based dietary choices (such as seeds, fruits, and flowers), this flying squirrel species is of special consideration, because it is an arboreal obligate folivore, mainly feeding on leaf parts (including buds, petioles, young leaves, and mature leaves) of various broadleaf trees (Lee et al., 1986; Kuo and Lee, 2003). It occupies a unique feeding niche in treetops, escaping pressures of both competition and predation in forest floor ecosystems (Coley and Barone, 1996). However, since tree leaves often contain complex carbohydrates and secondary metabolites (i.e., plant defensive chemicals, such as flavonoids, alkaloids, and tannins; Mithofer and Boland, 2012), folivorous mammals must have an efficient digestive system to gather nutrients and concurrently avoid toxins from their leaf-based diets (Coley and Barone, 1996). These dual challenges may be overcome with multitudinous functions provided by gut microbiota (Kohl et al., 2014, 2016), which probably confer rapid dietary adaptation (Alberdi et al., 2016). It is noteworthy that this flying squirrel species has a large population in wide montane regions of Taiwan and East Asia (Smith and Johnston, 2016), which implies successful feeding strategies, including effective gut microbiota. Thus, the flying squirrel's cecal microbiota should be an ideal model to investigate microbial functional characteristics with regard to a high-fiber, toxin-rich diet.

**Figure 1:**
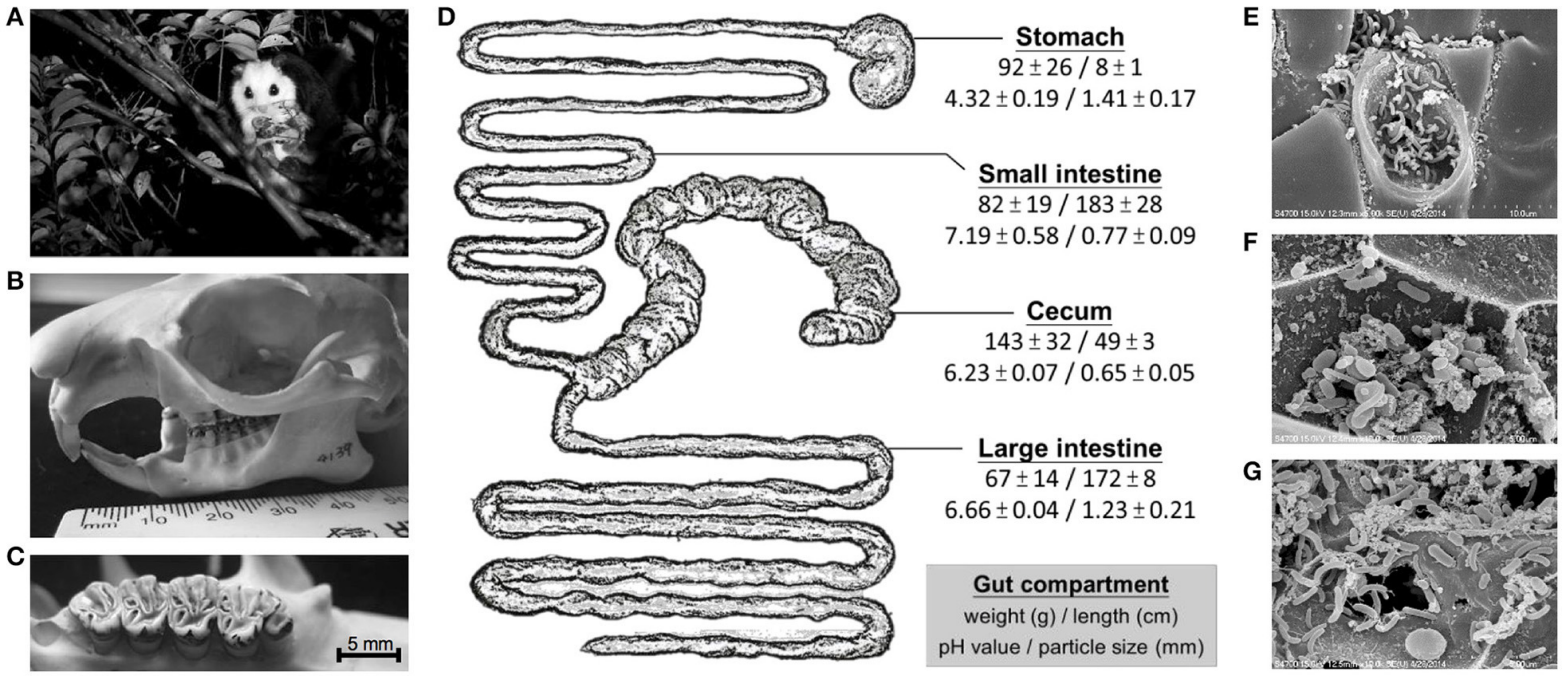
Digestive strategies of the leaf-eating flying squirrel. **(A)** White-faced flying squirrel (*Petaurista alborufus lena*) occupies a unique feeding niche in the treetop. **(B)** The skull structure (mouth with a broad gap) enables temporarily holding a large volume of tree leaves before chewing them. **(C)** Molars with multiple narrow folds on the crown enable tree leaves to be well chewed. **(D)** Anatomical/physiological characteristics (mean ± SD) of the four main gut compartments; note the extremely enlarged cecum which contained the majority of feed contents for microbial fermentation. **(E–G)** The cecum harbored large numbers of microorganisms that act on plant debris. Photo credits: **(A)** Hsueh-Chen Chen; **(B,C)** Ji-Fan Hsieh; and **(E–G)** Han-Chen Ho.

Objectives were to elucidate anatomical/physiological characteristics of the flying squirrel's digestive system and functional characteristics of its cecal microbiota under a high-fiber and toxin-rich diet. In our previous study using 16S rRNA gene libraries to investigate spatial heterogeneity of the flying squirrel's gut microbiota (Lu et al., 2014), we reported that the cecum, an enlarged part of the gut, contained relatively high bacterial diversity. Here, we further investigated this specialized system, using multiple “meta-omic” approaches, including metabolomics, metagenomics and metatranscriptomics (Segata et al., 2013; Aguiar-Pulido et al., 2016). Although each meta-omic approach has been widely used alone, few studies have integrated multiple meta-omic data to understand animal-microbe interactions. In this study, functional features of the cecal microbiota were profiled with three meta-omic approaches; this enabled complementary confirmation of microbial activities (Segata et al., 2013; Aguiar-Pulido et al., 2016). Specifically, we used metabolomes to trace the metabolic fate of dietary compounds along the digestive tract, and also used parallel metagenomes and metatranscriptomes to detect discrepancies between existing genes (DNA level; potential functions) and expressed genes (RNA level; realized functions) of the cecal microbiota. These meta-omic data were also compared to open-access gut metagenomes from other mammals to reveal unique characteristics of the flying squirrel's cecal microbiota. Moreover, to provide a general understanding of this system, we measured mass, pH, and feed particle size throughout the gut, and included images of flying squirrel's skull and teeth, as well as microscopic features of cecal microorganisms (Figure 1). This exploratory study provides findings which may help to form molecular-based hypotheses regarding functional contributions of symbiotic gut microbiota in small herbivores with folivorous dietary habits.

## Materials and methods

### Sample collection

Five white-faced flying squirrels (*Petaurista alborufus lena*) were captured from the mountains of Taiwan. A wild animal collecting permit (No. 0990007029) was approved by Yushan National Park Headquarters, Taiwan. Animals were dissected immediately after death. All experiments were performed in accordance with the Wildlife Conservation Act (http://law.moj.gov.tw/Eng/LawClass/LawAll.aspx?PCode=M0120001). All five individuals were used for measurements of gut structure and feed contents, whereas three were used for the metabolomes and the remaining two were used for metagenomes/metatranscriptomes.

### Gastrointestinal anatomy and physiology

The length and weight (with feed contents included) of the stomach, small intestine, cecum, and large intestine (i.e., four main gut compartments) were measured and reported (Lu et al., 2012). Here, we additionally examined the pH of feed contents. Moreover, to understand how plant-based diets were processed along the digestive tract, feed contents (~10 g from each gut compartment) were passed through the graded sieves (pore sizes: 4, 2, 1, 0.5, 0.25, and 0.125 mm) by wet-sieving (Clauss et al., 2002). Thereafter, feed particles of each size fraction (seven groups: > 4.0, 2–4, 1–2, 0.5–1, 0.25–0.5, 0.25–0.125, and <0.125 mm) were transferred onto a Petri dish, dried at 60°C for 24 h, and weighed after cooling to room temperature. Mean particle size of feed contents from each gut compartment was calculated by fitting a normal distribution (Fritz et al., 2009). Based on these measurements, we sketched the shape of the flying squirrel's digestive tract and marked physiologic characteristics of each gut compartment (Figure 1). In addition, for a visual image of gut microbes, feed contents from the cecum were fixed and observed with a Hitachi S-4700 field emission scanning electron microscope (FE-SEM). Specifically, specimens were pre-fixed with 2.5% glutaraldehyde in 0.1 M cacodylate buffer at 4°C, post-fixed with 1% osmium tetroxide in 0.1 M cacodylate buffer at room temperature, then dehydrated through a graded series of ethanol until they were in 100% ethanol, and then they were put into 100% acetone. Specimens were critical point dried, sputter coated with gold, and examined with a FE-SEM at 15 kV.

### Construction of metabolomes

For constructing metabolomes, feed contents from three individuals were frozen (−20°C), immediately after death. For each individual, a total of 15 samples from the stomach, small intestine, cecum, and large intestine (2, 5, 4, and 4, respectively; each ~0.5 g) were collected (Supplementary Figure 1). Each sample was homogenized with 3-fold distilled water for 5 min. The supernatant containing metabolites was retrieved by centrifugation (10,000 × g for 10 min) and mixed with 100% methanol (Sigma-Aldrich, CHROMASOLV®, for HPLC) at a ratio of 1:3, and repeated once. The supernatant was lyophilized and dissolved in 100 μl distilled water for metabolomic detection by liquid chromatography (LC)—electrospray ionization (ESI)—mass spectrometry (MS), composed of an ultra-performance LC (Ultimate 3000 RSLC, Dionex) coupled with an ESI source of quadrupled time-of-flight MS (maXis HUR-QToF system, Bruker Daltonics). Metabolites were separated by reversed-phase liquid chromatography on an HSS T3 C18 column (2.1 × 100 mm; Walters). The LC parameters were autosampler temperature at 4°C and injection volume of 10 μl with flow rate at 0.4 ml/min. Elution started from 99% mobile phase A (0.1% formic acid in pure water) and 1% mobile phase B (0.1% formic acid in ACN). Mobile phase B was held at 1% for 0.5 min, raised to 60% in 6 min, raised to 90% in 0.5 min and held for 1.5 min, and lowered to 1% in 5 min. Finally, the column was equilibrated by pumping 99% mobile phase A for 4 min. The LC–ESI–MS chromatograms were acquired by the following parameters: 190°C dry gas at 8 L/min flow rate, and 1.4 bar nebulizer gas with 4,500/3,500 capillary voltage for positive/negative ion modes. The m/z values in mass spectra were recorded for further data processing.

### Metabolomic profiles across gut compartments

Metabolomic data acquired from the LC–ESI–MS chromatograms were processed by TargetAnalysis (Version 1.1, Bruker Daltonics) and XCMS (Smith et al., 2006) with optimized parameters for Bruker Q-TOF mass spectrometer (Tautenhahn et al., 2008). Metabolites were identified by MetaboSearch (Zhou et al., 2012) and matched with theoretical m/z values against the Madison Metabolomics Consortium Database (Cui et al., 2008). Identified metabolites were further annotated according to the reference library of KEGG compounds (Hattori et al., 2003), with the tolerance of LC peaks within 0.3 min and signal intensities >1,000 counts. These compound annotations were used for profiling chemical environments of each gut compartment.

The overall similarity/dissimilarity among metabolomic profiles was assessed with Jaccard distance and displayed in an ordination diagram using principal coordinates analysis (PCoA), generated by the QIIME (Version 1.9) pipeline (Caporaso et al., 2010). Detected KEGG compounds were further grouped according to their chemical structures and cellular functions (Hattori et al., 2003), with an emphasis on the quantity and signal intensity of phytochemicals (leaf-derived compounds) across gut compartments. Multiple comparisons were done by ANOVA coupling with Scheffé's test, using “stats” and “agricolae” packages (Mendiburu, 2016) in R (R Development Core Team, 2016).

Finally, metabolomic data were compiled with metagenomic and metatranscriptomic data (described below) to detect potentially crucial functional reactions of cecal microbiota under the leaf-based diet.

### Construction of metagenomes and metatranscriptomes

For constructing metagenomes and metatranscriptomes, feed contents from two individuals were collected immediately after death and placed in RNAlater solution (Ambion, Life Technologies). For each individual, samples from the cecum (Supplementary Figure 1, sites 8–11, each ~5 g) were pooled to represent the entire cecal microbiota. Total DNA and RNA were isolated using the AllPrep DNA/RNA Mini Kit (QIAGEN), according to manufacturer's instructions. Briefly, cecal contents were centrifuged (10,000 × g for 10 min) to remove RNAlater and re-suspended in lysis buffer (TE buffer with 5 mg/mL lysozyme) at room temperature for 5 min. After addition of RLT buffer (with beta-mercaptoethanol), lysate was homogenized by passing it 10 times through a 20-G needle with a 1-ml syringe. The DNA molecules were purified through an AllPrep DNA spin column, and RNA molecules (>200 bp, without small rRNAs and tRNAs) were isolated with an RNeasy spin column with DNase treatment (QIAGEN). Furthermore, 16S rRNAs and 23S rRNAs (typically accounting for >80% of total RNA molecules) were removed using a MICROB*Express*™ Bacterial mRNA Enrichment Kit (Ambion, Life Technologies), according to the manufacturer's instructions. Quantity and quality of DNA and RNA samples were estimated using a NanoDrop 2000 Spectrophotometer (Thermo Scientific) and an Agilent 2100 bioanalyzer (Agilent Technologies).

Coupled DNA and RNA samples were subjected to shotgun sequencing on a GS-FLX Titanium system (Roche Life Science) for characterizing metagenomes and metatranscriptomes. For RNA samples, double-stranded cDNA libraries were constructed according to the cDNA Synthesis System Kit (Roche Life Science). The DNA and cDNA libraries were converted into single-stranded DNA fragments for sequencing using a GS-FLX Titanium Rapid Library Preparation Kit (Roche Life Science). Two full runs of shotgun sequencing were conducted. Sequence data were submitted to the NCBI Sequence Read Archive (SRA; BioProject accession PRJNA267179).

### Metagenomic and metatranscriptomic profiles of cecal microbiota

Raw reads of metagenome (DNA-based) and metatranscriptome (RNA-based) sequences were filtered to remove exact duplicates (possible artifacts from emulsion PCR) using CD-hits (Li and Godzik, 2006), and low-quality parts (Phred quality < 25, N content > 3%, sequences < 100 bp) were trimmed using SeqClean (http://seqclean.sourceforge.net). After quality control, a total of 569,349 metagenome reads and 483,241 metatranscriptome reads (both with average length > 300 bp) were used for bioinformatic analyses. Potential rRNA sequences were identified using BLASTn (cut-off e-value < 1e-5, with > 90% alignment) against the SILVA (containing eukaryotic and prokaryotic ribosomal RNA sequences) database (Pruesse et al., 2007). Remaining non-rRNA sequences were considered as mRNA reads for protein-coding gene annotation. Detailed sequence statistical information is shown in Supplementary Table 1.

To provide taxonomic profiles of metagenomes and metatranscriptomes, putative mRNA reads were mapped to NCBI-nr (non-redundant protein database of National Center for Biotechnology Information) database (Sayers et al., 2012) using BLASTX (cut-off e-value < 1e-5) to identify potential taxonomic origins. Taxonomic classification of sequences was retrieved and summarized according to NCBI Taxonomy. For domain—and phylum-level taxonomic profiles, all significant hits (cut-off e-value < 1e-5) were considered. For fine-level taxonomic identification, minimum identity thresholds of amino acid sequences were further restricted at 45 and 65% for family- and genus-level taxonomic groups, respectively (Konstantinidis et al., 2017).

To provide functional profiles of metagenomes and metatranscriptomes, FragGeneScan (Rho et al., 2010) based on the hidden Markov model was used to predict open reading frames (ORFs) on putative mRNA reads. The ORFs were queried to identify conserved protein families and domains with amino acid sequences against: (1) COG (Clusters of Orthologous Groups of proteins) database (Galperin et al., 2015); (2) Pfam (Protein families) database (Finn et al., 2016); and (3) KEGG (Kyoto Encyclopedia of Genes and Genomes) database (Kanehisa and Goto, 2000). Specifically, COG annotation was performed using a reverse position-specific BLAST algorithm (RPS-BLAST, cut-off e-value < 1e-5) against NCBI COG database (latest update 2017/3/28). Pfam annotation was performed using a HMMER3 (cut-off e-value < 1e-5) against Pfam database (version 31.0). In addition, to focus on carbohydrate-degrading enzymes, glycoside hydrolases (GHs) were determined according to correspondence between Pfam and CAZy (Carbohydrate-Active enZYmes; Cantarel et al., 2009). KEGG Orthology (KO) annotation was performed using KEGG GhostKOALA (Kanehisa et al., 2016), which is designed for metagenome sequences. Moreover, to reconstruct potentially crucial functional reactions conducted by the cecal microbiota, compounds, and genes detected in the metabolome, metagenome, or metatranscriptome were all mapped to KEGG modules (Takami et al., 2012) and pathways (Hattori et al., 2003).

To evaluate individual variation, Pearson correlation coefficients (*r*) were used to assess degree of similarity between results derived from two flying squirrel individuals, using the “stats” package of R (R Development Core Team, 2016).

### Meta-analysis of mammalian gut metagenomes

To provide an overview of functional characteristics of mammalian gut microbiota, in addition to 2 metagenome datasets from the flying squirrel's cecum, 4 metagenome datasets from cow's rumen (Brulc et al., 2009), and 39 metagenome datasets from fecal samples of zoo mammals (Muegge et al., 2011) were downloaded and analyzed. All datasets were processed with the same procedures for the ORF prediction and functional annotation, as mentioned above. Overall similarity/dissimilarity of mammalian gut metagenomes was assessed with Bray-Curtis dissimilarity and displayed in an ordination diagram using nonmetric multidimensional scaling (NMDS). An ANOSIM was done to determine whether there were significant differences in functional profiles (based on COG or Pfam) among five metagenome groups (i.e., flying squirrel's cecum, cow's rumen, zoo carnivores' feces, zoo omnivores' feces, and zoo herbivores' feces). Moreover, we performed a Kruskal-Wallis test with a threshold of adjusted *p*-value (FDR) <0.05 to assess significant gene families and detect different relative abundances among the five metagenome groups. Then, Dunn's test was used as a post-hoc procedure for pairwise multiple comparisons to detect the uniqueness of the flying squirrel's cecal metagenome, compared to all other gut metagenomes. Those statistical assessments were conducted using “vegan” (Oksanen et al., 2017), “RVAideMemoire” (Hervé, 2017), and “stats” packages in R (R Development Core Team, 2016).

## Results

### Anatomical/physiological characteristics of the flying squirrel's digestive system

The leaf-eating flying squirrel (Figure 1A) had a skull and molars (Figures 1B,C) that were specialized, facilitating mastication of tree leaves. In addition, shape and size both varied substantially across four main gut compartments, with the cecum being the largest chamber (Figure 1D). The pH of feed contents also differed among gut chambers (Figure 1D), with stomach contents having a relatively low pH (4-5), small intestine contents being slightly alkaline (pH 7-8), and contents in the cecum and large intestine being mildly acidic (both pH 6-7). Regarding feed particle sizes (Figure 1D and Supplementary Figure 2), feed particles in the stomach were already fine (on average, ~1.5 mm). Nevertheless, compared to dry and coarse fibers present in the stomach, feed contents in the cecum were even finer (on average, ~0.6 mm) with a sludge-like texture (Supplementary Figure 3). Moreover, in microscopic evaluation of cecal contents, various microbial cells were closely attached to plant debris (Figures 1E–G).

### Metabolomic compositions across the flying squirrel's gut compartments

**Figure 2:**
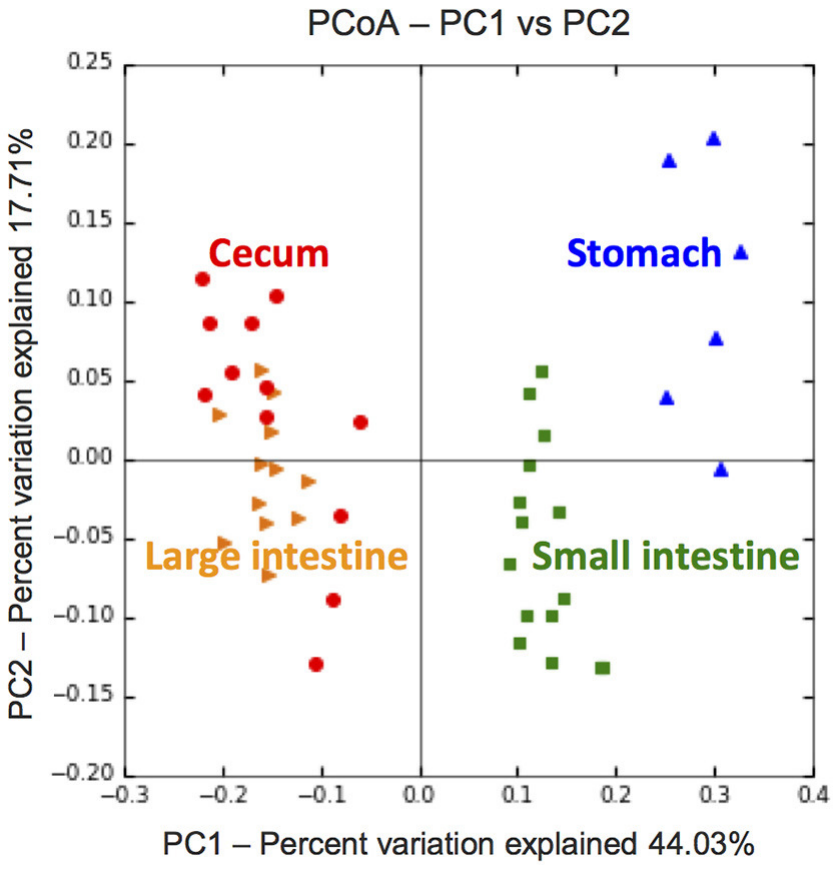
PCoA plot of metabolomic compositions revealing distinct chemical environments across gut compartments of the flying squirrel (*N* = 3). For each individual, 2, 5, 4, and 4 metabolomic samples were collected from the stomach, small intestine, cecum, and large intestine, respectively (see Figure S1 for details).

**Figure 3:**
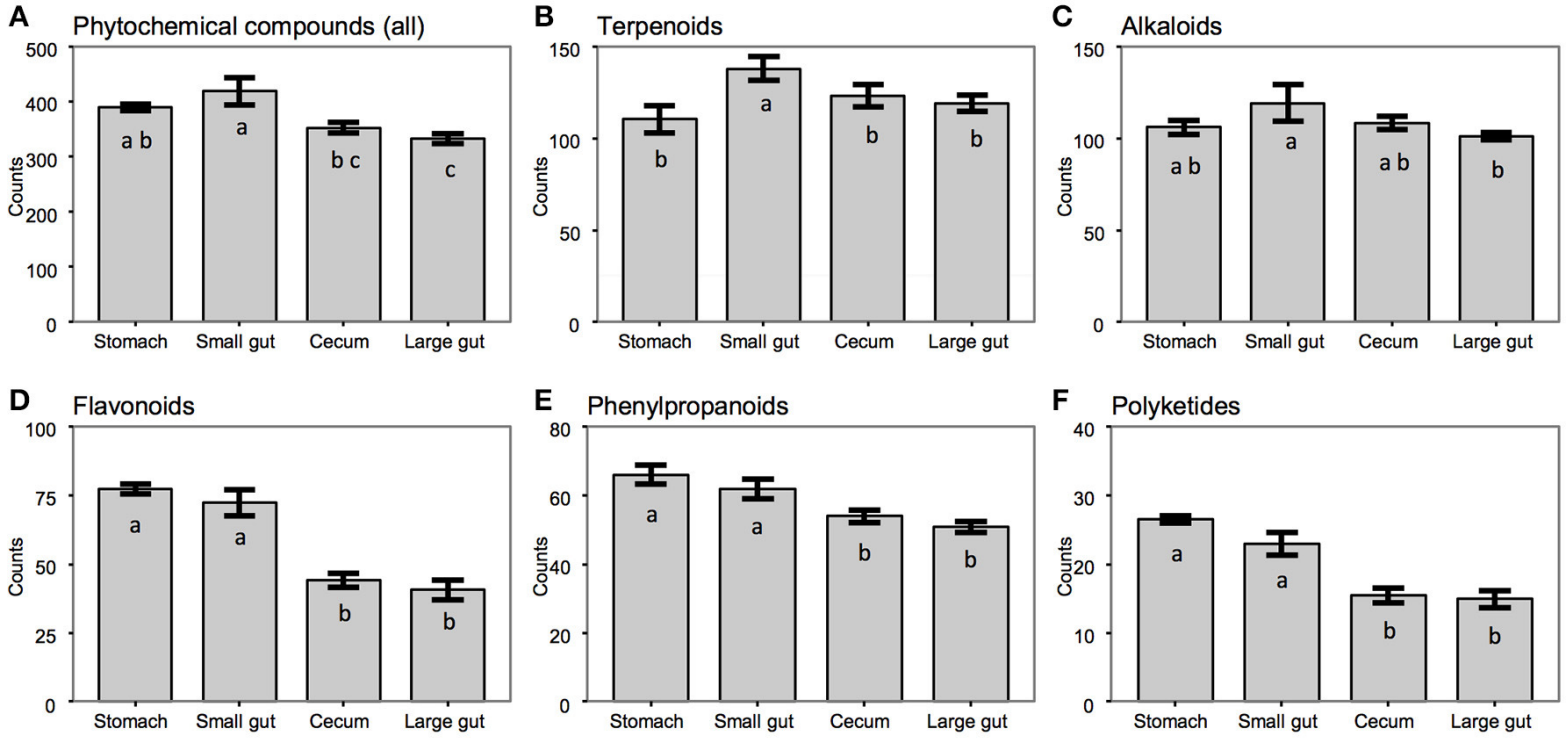
Numbers of phytochemical compounds determined across gut compartments of the flying squirrel (*N* = 3), for **(A)** entire group of phytochemicals or **(B–F)** each subgroup. ^a–c^Columns without a common letter differed (*P* < 0.05); error bars are standard error of the mean (SEM) for number of compounds detected within each gut region.

Based on LC–ESI–MS metabolomic profiles, each gut compartment contained its own unique compound composition in feed contents, with clear differences between samples from the upper (stomach and small intestine) vs. lower (cecum and large intestine) portions of the digestive tract (Figure 2). Overall, there were ~600 phytochemicals (mainly leaf-derived metabolites) detected among all gut samples. Several phytochemicals (~20%) were present only in the stomach and small intestine, with relatively fewer phytochemicals detected in the cecum and large intestine (Figure 3), especially flavonoids, phenylpropanoids, and polyketides (Figures 3D–F). Moreover, considering the relative intensity of those detected phytochemicals, their signal intensities were also much lower in the hindgut than in the foregut (Supplementary Figure 4).

### Metagenomes and metatranscriptomes of the flying squirrel's cecal microbiota

**Figure 4:**
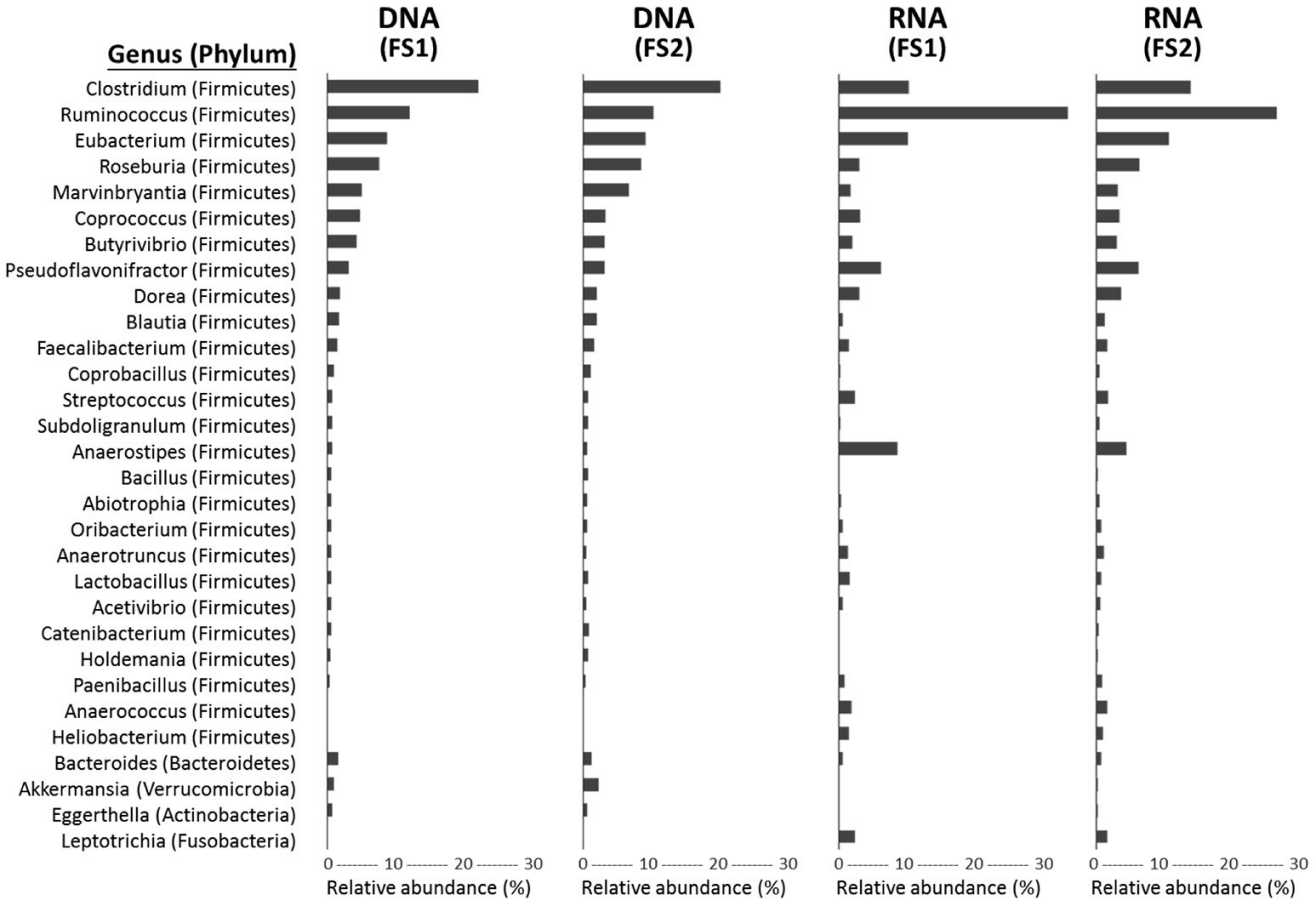
Genus-level taxonomic compositions of metagenomes (DNA-level) and metatranscriptomes (RNA-level) of cecal microbiota from two flying squirrels (FS1 and FS2). Top 30 abundant genera that constituted >0.5% in either library are shown, with their phyla in parentheses.

Based on the annotation of protein-coding sequences, at both DNA and RNA levels, cecal microbiota were dominated by bacteria (average, 97.94% of DNA reads and 88.24% of RNA reads; Supplementary Table 2), followed by eukaryotes (0.77 and 11.37%), archaea (0.46 and 0.11%), and viruses (0.09 and 0.03%). Targeting bacterial composition, the phylum Firmicutes was extremely abundant in both metagenomes (~90%) and metatranscriptomes (~85%), with Actinobacteria, Proteobacteria, Bacteroidetes constituting only a small fraction (Supplementary Table 3). At a genus level, 577 bacterial taxa were detected, whereas only 30 taxa contained >0.5% reads in any library (Figure 4). Among these abundant genera, *Clostridium, Ruminococcus*, and *Eubacterium* (all belonging to Firmicutes) were the top three in all libraries, contributing to the majority (up to 50%) of annotated protein-coding genes in the cecal metagenome and metatranscriptome.

**Figure 5:**
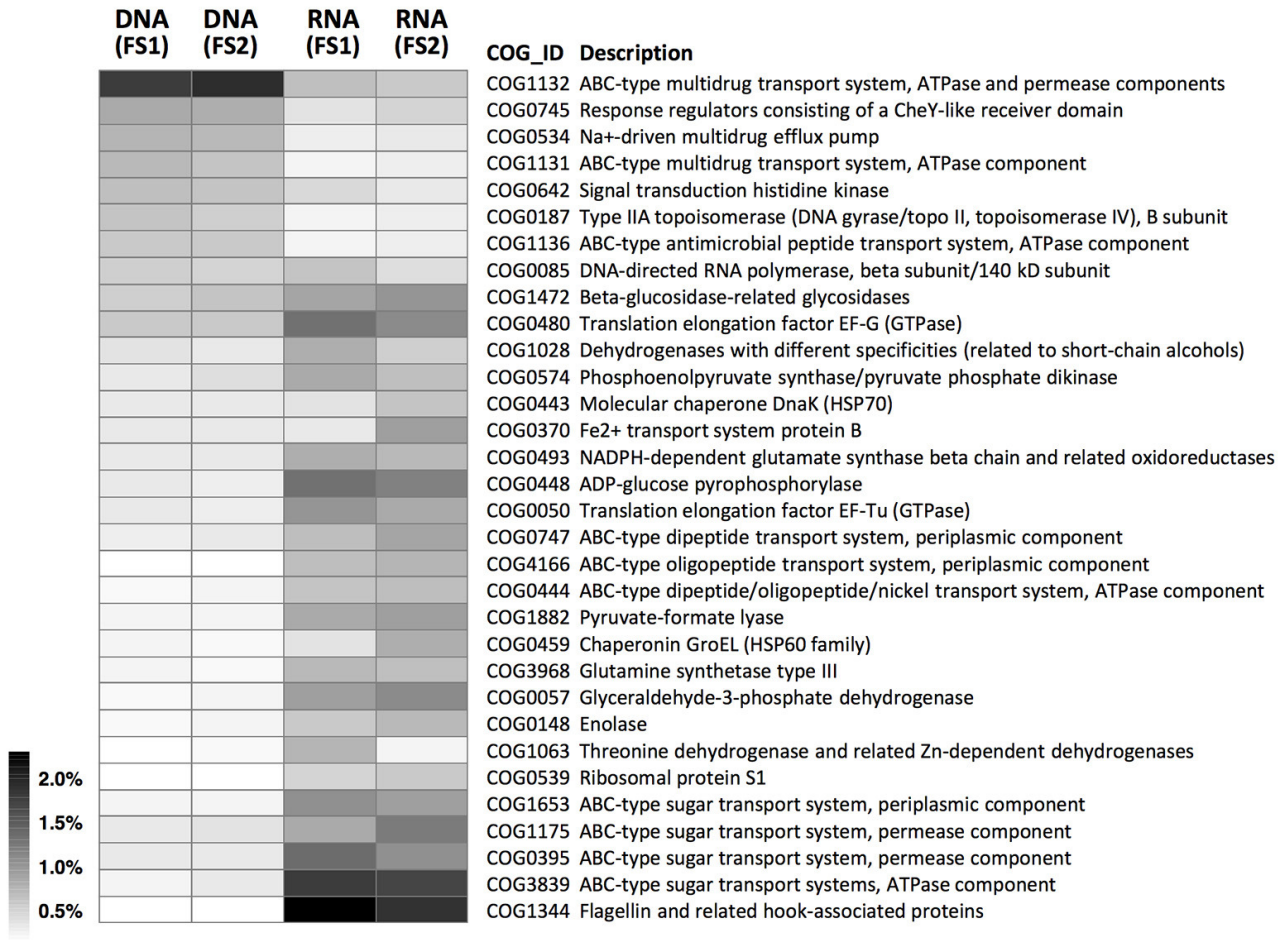
Abundance distributions of COGs in metagenomes (DNA-level) and metatranscriptomes (RNA-level) of cecal microbiota from two flying squirrels (FS1 and FS2). Only abundant COGs that constituted >0.5% in either library are shown.

According to the COG annotation (Figure 5 and Supplementary Figure 5), functional gene families had distinct abundance patterns at DNA and RNA levels. Specifically, the most abundant COG in the cecal metagenome was assigned to the ABC-type multidrug exporter component (COG1132; involved in defense mechanisms; representing ~2% of hits; Figure 5). By contrast, the most abundant COG in the cecal metatranscriptome was assigned to the flagellin protein (COG1344; involved in cell motility), followed by several ABC-type sugar importer components (e.g., COG3839; involved in carbohydrate transport and metabolism). In addition, ABC-type importer components for short peptides (e.g., COG0747, involved in amino acid transport and metabolism) also had relatively high abundances at the RNA level.

**Figure 6:**
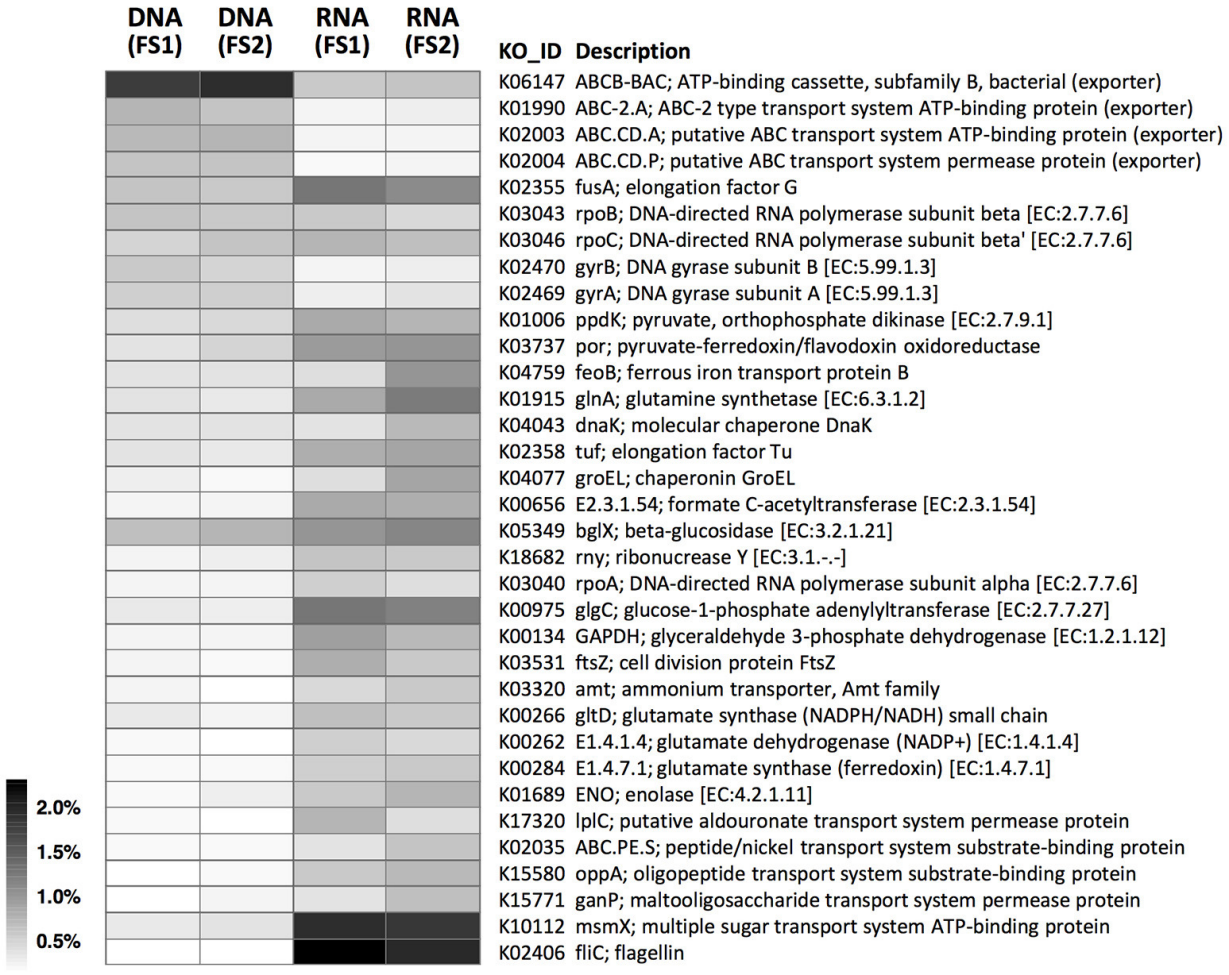
Abundance distributions of KOs in metagenomes (DNA-level) and metatranscriptomes (RNA-level) of cecal microbiota from two flying squirrels (FS1 and FS2). Only abundant KOs that constituted >0.5% in either library are shown.

Contrasting functional gene profiles in the metatranscriptome vs. metagenome were also revealed by KEGG annotation (Figure 6 and Supplementary Figure 6), with distinct types of membrane transport components dominant at RNA—vs. DNA-levels, similar to COG results (Figures 5, 6). In combination with metabolomic data, we identified KEGG pathways with high coverage of KOs and relevant compounds, including pathways involved in substrate-induced cell motility (i.e., bacterial chemotaxis and flagellar assembly; Supplementary Figures 7, 8) and pathways for biosynthesis of cellular components from plant-sourced nutrients (e.g., cellobiose, xylose, and arabinose were transported into bacterial cells and converted into other macromolecules, such as amino acids, nucleotides, and peptidoglycans; Supplementary Figures 9–16).

**Figure 7:**
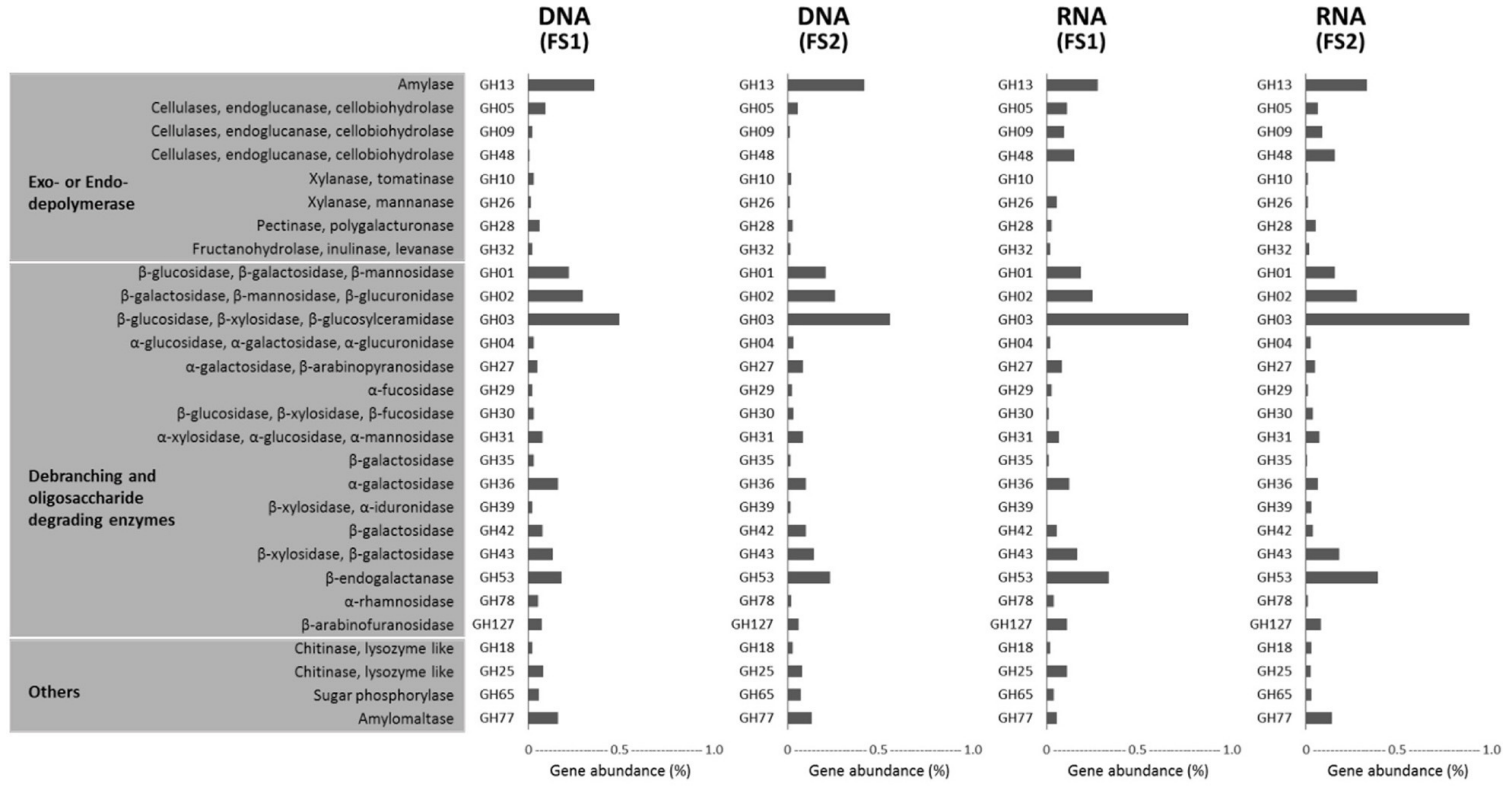
Glycoside hydrolases (GHs) detected in metagenomes (DNA-level) and metatranscriptomes (RNA-level) of cecal microbiota from two flying squirrels (FS1 and FS2). Only abundant GHs that constituted >0.05% in either library are shown.

Among those abundantly detected COGs and KOs (Figures 5, 6), it was notable that apart from ABC-type transport systems and genes associated with “housekeeping functions” (such as DNA gyrases, RNA polymerases, and translation elongation factors), one particular carbohydrate-degrading enzyme, beta-glucosidase (COG1472 or K05349), had high abundance in both metagenomes (~0.5%) and metatranscriptomes (~1.0%). To better characterize diversity of carbohydrate-degrading enzymes, we focused on glycoside hydrolase (GH) groups, based on the Pfam annotation. A total of 60 GH groups were detected at the DNA level, of which, 39 GH groups were also detected at the RNA level (Supplementary Table 4). According to enzymatic activities of those GH groups, diverse carbohydrate-degrading genes specific for beta-glycosidic linkages in plant polysaccharides / oligosaccharides were detected in the cecal metagenome and metatranscriptome (Figure 7). Among them, GH3 was the most abundant group at both DNA and RNA levels. Moreover, compared to the DNA-level background, GH9, GH48, GH43, and GH53 were enriched at the RNA level (Figure 7).

Basically, taxonomic and functional profiles from individuals (FS1 and FS2) were comparable (Figures 4–7), with a high similarity (*r* > 0.9 and *P* < 0.01) for either metagenomes or metatranscriptomes.

### Meta-analysis of mammalian gut metagenomes

**Figure 8:**
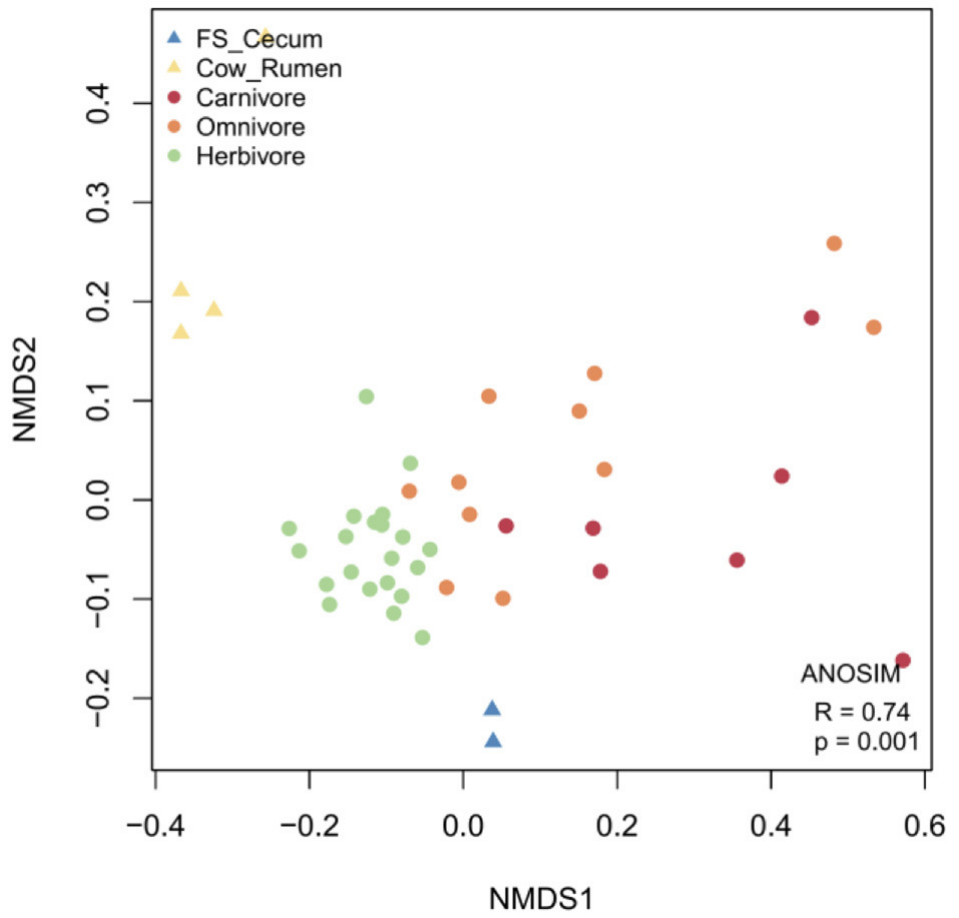
NMDS plot based on COG compositions, revealing functional similarity / dissimilarity among mammalian gut metagenomes. In addition to two datasets from the flying squirrel (FS) cecum of this study, publicly available gut metagenomes, including four datasets from the cow's rumen and 39 datasets from the fecal samples of zoo mammals (categorized by diets: carnivore, omnivore, and herbivore), were included for comparison.

To provide an overview of functional characteristics of mammalian gut microbiota, in addition to 2 metagenomes from the flying squirrel's cecum, we conducted a meta-analysis on mammalian gut metagenomes with published datasets (including four datasets from the cow's rumen, and 39 datasets from fecal samples of zoo carnivores/omnivores/herbivores). Functional gene compositions based on the COG annotation (Figure 8) demonstrated that: (1) functional profiles of cecal metagenomes and ruminal metagenomes were distinct from those based on fecal samples; and (2) functional profiles of cecal metagenomes were also distinct from those of ruminal metagenomes. Similar functional relationships were obtained based on the Pfam annotation (Supplementary Figure 17).

To reveal unique functional characteristics of the flying squirrel's cecal microbiota, we conducted statistical tests among 5 metagenome groups (i.e., flying squirrel's cecum, cow's rumen, zoo carnivores' feces, zoo omnivores' feces, and zoo herbivores' feces). In comparisons based on relative abundances of COG functional categories, many categories had significant differences among 5 metagenome groups (Supplementary Table 5). Moreover, post-hoc pairwise comparisons focusing on specific differences between the flying squirrel's cecum and the other four groups (Supplementary Table 6) indicated that, despite several significant pairs, the flying squirrel's cecum had clear differences from all other groups in one particular category, namely defense mechanisms (V). Specifically, compared to other mammalian gut metagenomes, the flying squirrel's cecal metagenome contained a significantly higher percentage of gene function involved in defense mechanisms (Supplementary Table 5), especially for those COG gene families assigned to multidrug efflux pumps (Supplementary Table 7).

In addition, in a comparison of glycoside hydrolase (GH) groups among all 5 metagenome groups, the flying squirrel's cecal metagenome contained relatively high levels of GH3, GH43, and GH53 (Supplementary Table 8); those GH groups were specific for degradation of oligosaccharides and were actually enriched in cecal metatranscriptions (Figure 7).

## Discussion

In this study, we demonstrated specialized digestive strategies of the white-faced flying squirrel (*Petaurista alborufus lena*), including powerful molars to facilitate mastication of tree leaves and an extended cecum to store ingesta for microbial activities (Figure 1). Notably, size distribution of feed particles in the large intestine was more similar to that in the stomach than in the cecum (Figure 1D and Supplementary Figure 2), indicating that some large feed particles might go directly to the large intestine (bypassing the cecum), corresponding to the description of specialized sorting at the ileal-cecal-colic junction in small herbivores (Sakaguchi, 2003). Overall, based on these anatomical/physiological characteristics, we inferred that transformation of dietary compounds into absorbable nutrients would primarily be conducted in the cecum with the aid of microbial activities.

Based on mass spectrometry to detect metabolites in feed contents, distinct gut compartments contained various types/levels of compounds (Figure 2). More importantly, many phytochemicals (leaf-derived compounds) were present only in the stomach and small intestine, but were not detected in the cecum and large intestine (Figure 3). We inferred that those phytochemicals were likely released from plant cells following exposure to a low pH and enzymes in the foregut, and later degraded or transformed by gut microbes in the hindgut (Deprez et al., 2000; Williamson et al., 2000). Some phytochemicals with sugar moieties could be used by gut microbes (Cardona et al., 2013), as a key factor structuring symbiotic microbial communities (Patra and Saxena, 2009; Laparra and Sanz, 2010; Ni et al., 2015). However, many phytochemical derivatives might be unwanted by bacterial cells (Cowan, 1999). We found that the flying squirrel's cecal metagenome contained various types of multidrug exporter genes with high abundances (Figures 5, 6), which may enable gut microbes to confer resistance to leaf-derived compounds or host-defense molecules (Neyfakh, 1997; Putman et al., 2000; Piddock, 2006). Alternatively, those efflux pumps may have other roles relevant to bacterial behavior in the cecal environment, such as quorum sensing and cell motility (Martinez et al., 2009; Buckner et al., 2016). Further studies are required to determine targets and roles of those genes.

Based on metagenomes and metatranscriptomes, the flying squirrel's cecal microbial communities were exceedingly dominated by bacterial Firmicutes taxa, including diverse genera (Figure 4). These taxonomic profiles based on protein-coding sequences were consistent with our previous results based on 16S rRNA gene sequences (Lu et al., 2014), suggesting that these Firmicutes taxa in general have adapted to the flying squirrel's cecum and contribute to major microbial activities in the gut environment.

We detected up to 60 types of glycoside hydrolases in the flying squirrel's cecal metagenome (Supplementary Table 4). This high diversity of GH groups was comparable to the diversity detected in other mammalian herbivores (Brulc et al., 2009; Flint et al., 2012; Wang et al., 2013; Jose et al., 2017), whereas detailed combinations and relative abundances of GH groups seemed to differ among animals with distinct gut structures and dietary preferences (Supplementary Table 8). For example, there were relatively high abundances of GH3, GH43, and GH53 in the flying squirrel's cecal metagenome, with even enriched levels in the cecal metatranscriptome (Figure 8). Despite different substrate specificities, these three dominant GH groups are involved in the degradation of beta-linkage oligosaccharides (Cantarel et al., 2009), releasing various monosaccharides. It is noteworthy that these GH enzymes may also have important roles in the degradation of phytochemicals (Deprez et al., 2000; Williamson et al., 2000), especially phenolic metabolites (e.g., flavonoids) that typically contain a sugar glycosidic linkage. That probably accounted for decreasing flavonoid compounds in the cecum (Figure 3). More importantly, those GH sequences contained a high nucleotide variation, and in fact, were assigned to various bacterial taxa (mostly belonging to Firmicutes, as listed in Figure 4), suggesting the importance of those enzymatic activities for microbes living in the flying squirrel's cecum.

**Figure 9:**
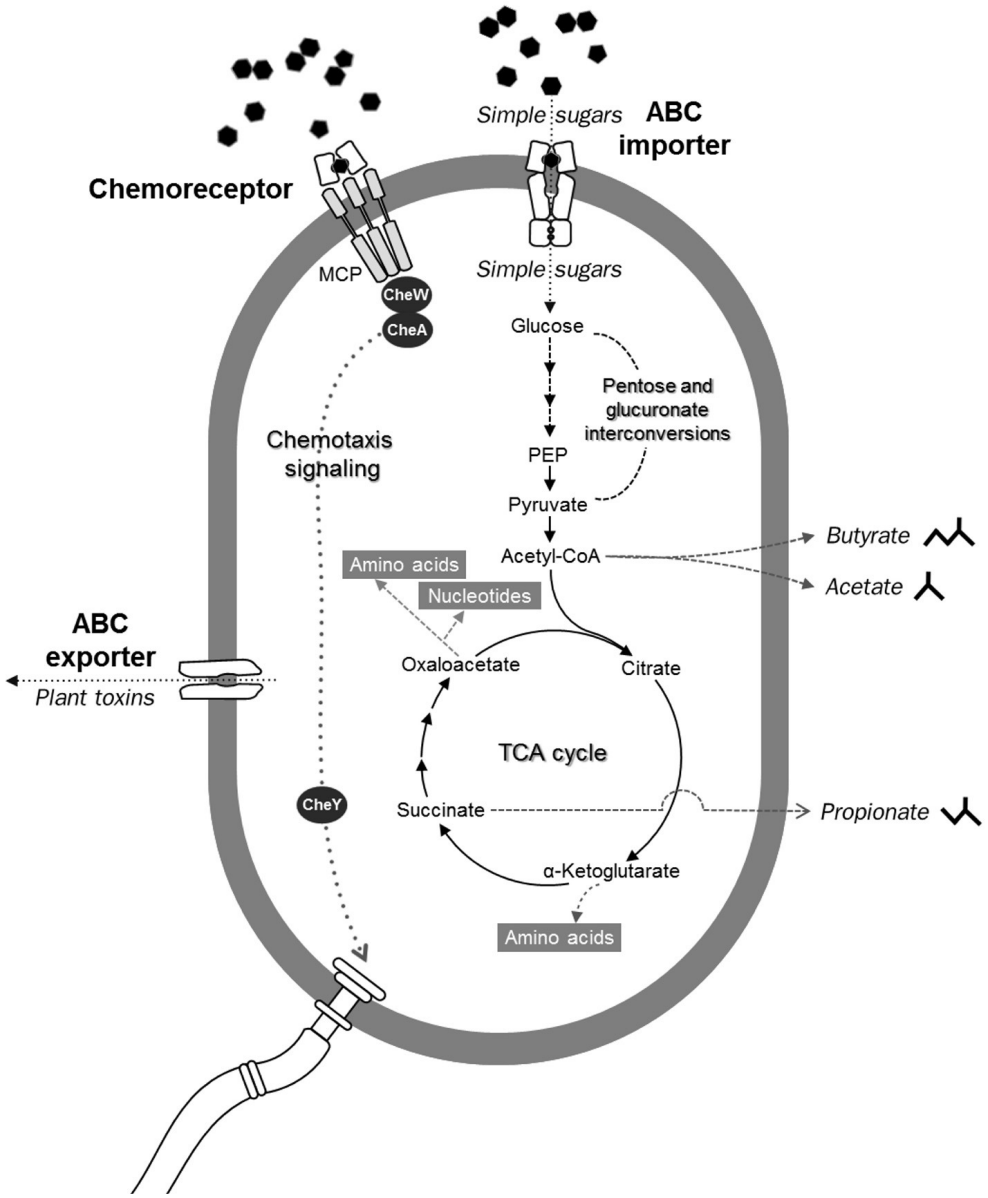
Diagrammatic illustration of potentially crucial microbial functions in the flying squirrel's cecum, based on genes and compounds detected in the metagenome, metatranscriptome, and metabolome. From top to bottom: simple sugars were transported into microbial cells through various ABC importers, and subsequently fermented into short-chain fatty acids. Meanwhile, substrate-binding components of ABC importers were also involved in chemotaxis signaling, enabling bacteria to move toward higher concentrations of sugars by flagellar motility. Left: ABC exporters involved in defense mechanisms may enable gut microbes to export toxic phytochemical derivatives.

In addition to carbohydrate-degrading ability (Figure 8), several genes involved in ABC-type sugar importers (Supplementary Figure 9) were detected in the flying squirrel's cecal microbiota, with many up-regulated at the RNA level (Figures 5, 6). Based on these findings, we inferred that the cecal microbiota may have a substantial ability to quickly transport simple sugars into cells, after investing enzymes for hydrolyzing plant fibers into monosaccharides (Figure 9; upper part). Moreover, pathways involved in substrate-induced cell motility (Di Paola et al., 2004), including chemotaxis signaling (Supplementary Figure 7) and flagellar structure (Supplementary Figure 8), were detected in both metagenomes and metatranscriptomes, indicating directional cell movement (i.e., sensing the gradient of surrounding nutrients and moving toward stimulatory chemicals; Figure 9; top to bottom of left side) were likely important for cecal microbiota. Notably, chemotaxis signaling and ABC-type transport systems have overlapping components (Figure 9; upper part); that is, the substrate-binding proteins of the ABC-type imports (Supplementary Figure 9) also mediate environmental stimuli for chemotaxis receptors (i.e., methyl-accepting chemotaxis protein, MCP; Supplementary Figure 7; Neumann et al., 2010). Thus, when sugar-binding genes were highly expressed (Figures 5, 6), it may not only allow microbes to quickly import sugars but also assist them to move toward sugar-rich microhabitats, thereby optimizing sugar acquisition in the cecum.

After being transported into the bacterial cell, sugar monomers (such as glucose, xylose, and arabinose) would be fermented into short-chain fatty acids (e.g., butyrate, acetate, and propionate) and subsequently converted into other cellular components (Figure 9; top to bottom of right side). In the cecal metagenome and metatranscriptome, pathways regarding pentose and glucuronate interconversions (Supplementary Figure 10), energy generating from sugar fermentation (Supplementary Figures 11, 12), and biosynthesis of various macromolecules (Supplementary Figures 13–16) were well-represented, indicating that plant-sourced sugars could be efficiently transformed into bacterial biomass in the flying squirrel's cecum, with adequate fermentation end-products to meet host requirements (Bergman, 1990; Stevens and Hume, 1998).

Our interpretation about functional characteristics of the cecal microbiota was based on the consistent patterns of two flying squirrel individuals, which may not be enough to robustly demonstrate how symbiotic microbes respond to unique environmental conditions in the cecum. Nevertheless, this study pointed out the importance of investigations into gut microbiota of small mammalian herbivores. Future research focusing on more individuals from leaf-eating flying squirrels and other small mammalian herbivores is demanded to capture the functional uniqueness of the cecal microbiota, considering different feeding adaptations and gut structures of large vs. small mammalian herbivores (Stevens and Hume, 1995; Mackie, 2002).

In the present study, we delineated anatomical/physiological characteristics of the flying squirrel's digestive system (Figure 1) and demonstrated functional characteristics of cecal microbiota based on multiple meta-omic data, including metabolomic profiles (Figures 2, 3) and parallel metagenome-metatranscriptome profiles (Figures 4–8). Here, we summarize crucial metabolic capacities of the flying squirrel's cecal microbiota (Figure 9), including the ability to: (1) secrete various glycoside hydrolases to degrade plant fibers into simple sugars; (2) transport simple sugars into the cells while also moving toward sugar-rich microhabitats; and (3) ferment sugars into short-chain fatty acids to generate energy for synthesizing amino acids and nucleotides. Moreover, compared to other mammalian gut metagenomes, the cecal metagenome of the flying squirrel tends to contain more multidrug exporter genes, which may enable gut microbes to export leaf-derived compounds. This study provided a molecular basis to promote understanding functional characteristics of symbiotic gut microbiota of small mammals with folivorous dietary habits in the wild.

## Data accessibility

NCBI Sequence Read Archive: SRX2118787—SRX2118794.

## Author contributions

H-PL, P-YL, and H-TY conceived the study design and collected samples; H-PL, P-YL, J-FH, S-WH, and H-CH conducted experiments; H-PL, P-YL, YW, and C-YL conducted bioinformatics analyses; H-PL, CH, and H-TY wrote the first draft. All authors contributed to data interpretation and preparation of the final manuscript. All authors reviewed and approved the final manuscript.

### Conflict of interest statement

The authors declare that the research was conducted in the absence of any commercial or financial relationships that could be construed as a potential conflict of interest.
