# Supplementary material for: Functional Characteristics of the Flying Squirrel's Cecal Microbiota under a Leaf-Based Diet, Based on Multiple Meta-Omic Profiling
Source: Front Microbiol. 2018 Jan 4;8:2622. doi: 10.3389/fmicb.2017.02622 (PMC5758534; doi:10.3389/fmicb.2017.02622)
Supplement: Supplementary file 1 [file Table1.pdf]

Supplementary Tables

Functional characteristics of the flying squirrel's cecal microbiota under a leaf-based diet, based on multiple meta-omic profiling

Hsiao-Pei Lu[1,#], Po-Yu Liu[1,2], Yu-bin Wang[1,3], Ji-Fan Hsieh[1,§], Han-Chen Ho[4], Shiao-Wei Huang[1], Chun-Yen Lin[3], Chih-hao Hsieh[1,5,6.7], and Hon-Tsen Yu[1,2,*]

[1]Department of Life Science, National Taiwan University, Taipei, Taiwan

[2]Genome and Systems Biology Degree Program, National Taiwan University & Academia Sinica, Taipei, Taiwan

[3]Institute of Information Science, Academia Sinica, Taipei, Taiwan

[4]Department of Anatomy, Tzu Chi University, Hualien, Taiwan

[5]Institute of Oceanography, National Taiwan University, Taipei, Taiwan

[6]Institute of Ecology and Evolutionary Biology, National Taiwan University, Taipei, Taiwan

[7]National Center for Theoretical Sciences, Taipei, Taiwan

[#]Present address: Institute of Oceanography, National Taiwan University, Taipei, Taiwan

[§]Present address: Division of Ecology and Evolution, Research School of Biology, The Australian National University, Canberra, Australia

[*]Correspondence:

Hon-Tsen Yu

E-mail: ayu@ntu.edu.tw

Supplementary Table S1. Sequence statistics of cecal metagenomes (DNA-level) and metatranscriptomes (RNA-level) of two flying squirrels (FS1 and FS2).

	FS1_DNA	FS2_DNA	FS1_RNA	FS2_RNA
Number of raw reads	331001	367615	282340	340122
Number of qualified reads	267265	302084	220614	262627
Average read length (bp)	315	303	346	334
Total library size (Mb)	84.19	91.5	76.34	87.79
Number of rRNA reads (SILVA)	939	927	126100	134670
(% of rRNA in total reads)	(0.35)	(0.31)	(57.16)	(51.28)
Number of non-rRNA reads	266326	301157	94514	127957
(% of non-rRNA in total reads)	(99.65)	(99.69)	(42.84)	(48.72)

Supplementary Table S2. Domain-level taxonomic classification for cecal metagenomes (DNA-level) and metatranscriptomes (RNA-level) of two flying squirrels (FS1 and FS2).

	FS1_DNA (%)	FS2_DNA (%)	FS1_RNA (%)	FS2_RNA (%)
Bacteria	171253 (97.98)	187950 (97.89)	62854 (88.26)	81578 (88.21)
Eukaryota	1305 (0.75)	1499 (0.78)	8083 (11.35)	10527 (11.38)
Archaea	783 (0.45)	911 (0.47)	51 (0.07)	128 (0.14)
Viruses	135 (0.08)	189 (0.10)	24 (0.03)	25 (0.03)
Unclassified	1306 (0.75)	1454 (0.76)	202 (0.28)	224 (0.24)

Supplementary Table S3. Taxonomic classification of Bacteria (at the phylum level) for cecal metagenomes (DNA-level) and metatranscriptomes (RNA-level) of two flying squirrels (FS1 and FS2).

	FS1_DNA (%)	FS2_DNA (%)	FS1_RNA (%)	FS2_RNA (%)
Firmicutes	153326 (89.53)	165414 (88.01)	53104 (84.49)	70023 (85.84)
Actinobacteria	4165 (2.43)	4665 (2.48)	1609 (2.56)	1764 (2.16)
Proteobacteria	3658 (2.14)	4037 (2.15)	4177 (6.65)	4399 (5.39)
Bacteroidetes	2256 (1.32)	2773 (1.48)	277 (0.44)	612 (0.75)
Tenericutes	1533 (0.90)	2681 (1.43)	82 (0.13)	331 (0.41)
Spirochaetes	1488 (0.87)	1765 (0.94)	169 (0.27)	428 (0.52)
Fusobacteria	1034 (0.60)	1333 (0.71)	1518 (2.42)	1381 (1.69)
Verrucomicrobia	951 (0.56)	1930 (1.03)	27 (0.04)	45 (0.06)
Synergistetes	518 (0.30)	649 (0.35)	58 (0.09)	134 (0.16)
Chloroflexi	329 (0.19)	341 (0.18)	33 (0.05)	68 (0.08)
Cyanobacteria	329 (0.19)	359 (0.19)	20 (0.03)	48 (0.06)
Thermotogae	295 (0.17)	456 (0.24)	18 (0.03)	104 (0.13)
Aquificae	132 (0.08)	125 (0.07)	257 (0.41)	195 (0.24)
Deinococcus-Thermus	114 (0.07)	101 (0.05)	294 (0.47)	147 (0.18)
Acidobacteria	70 (0.04)	101 (0.05)	1059 (1.68)	1535 (1.88)
Others (< 0.1%)	1055 (0.62)	1220 (0.65)	152 (0.24)	364 (0.45)

Supplementary Table S4. Abundance distributions of glycoside hydrolase (GH) groups detected in cecal metagenomes (DNA-level) and metatranscriptomes (RNA-level) of two flying squirrels (FS1 and FS2).

	FS1_DNA	(%)	FS2_DNA	(%)	FS1_RNA	(%)	FS2_RNA	(%)
GH_1	198	(0.18)	208	(0.17)	20	(0.15)	38	(0.13)
GH_2	269	(0.24)	261	(0.21)	27	(0.20)	66	(0.22)
GH_3	446	(0.40)	559	(0.45)	70	(0.52)	214	(0.71)
GH_4	25	(0.02)	33	(0.03)	2	(0.01)	6	(0.02)
GH_5	85	(0.08)	54	(0.04)	12	(0.09)	16	(0.05)
GH_6	3	(0.00)	1	(0.00)	0	(0.00)	0	(0.00)
GH_8	4	(0.00)	0	(0.00)	0	(0.00)	0	(0.00)
GH_9	20	(0.02)	10	(0.01)	10	(0.07)	15	(0.05)
GH_10	24	(0.02)	20	(0.02)	0	(0.00)	2	(0.01)
GH_13	326	(0.29)	419	(0.34)	30	(0.22)	80	(0.27)
GH_14	1	(0.00)	0	(0.00)	2	(0.01)	1	(0.00)
GH_15	1	(0.00)	4	(0.00)	0	(0.00)	0	(0.00)
GH_16	4	(0.00)	16	(0.01)	0	(0.00)	0	(0.00)
GH_18	19	(0.02)	28	(0.02)	2	(0.01)	7	(0.02)
GH_20	6	(0.01)	6	(0.00)	1	(0.01)	0	(0.00)
GH_24	3	(0.00)	4	(0.00)	1	(0.01)	0	(0.00)
GH_25	73	(0.06)	80	(0.06)	12	(0.09)	6	(0.02)
GH_26	13	(0.01)	12	(0.01)	6	(0.04)	2	(0.01)
GH_27	44	(0.04)	82	(0.07)	9	(0.07)	12	(0.04)
GH_28	55	(0.05)	28	(0.02)	3	(0.02)	13	(0.04)
GH_29	17	(0.02)	24	(0.02)	3	(0.02)	2	(0.01)
GH_30	24	(0.02)	30	(0.02)	1	(0.01)	9	(0.03)
GH_31	69	(0.06)	84	(0.07)	7	(0.05)	17	(0.06)
GH_32	18	(0.02)	14	(0.01)	2	(0.01)	4	(0.01)
GH_35	24	(0.02)	14	(0.01)	1	(0.01)	1	(0.00)
GH_36	143	(0.13)	98	(0.08)	13	(0.10)	16	(0.05)
GH_37	0	(0.00)	3	(0.00)	0	(0.00)	0	(0.00)
GH_38	16	(0.01)	17	(0.01)	0	(0.00)	1	(0.00)
GH_39	18	(0.02)	17	(0.01)	0	(0.00)	7	(0.02)
GH_42	69	(0.06)	100	(0.08)	6	(0.04)	9	(0.03)

GH_43	163 (0.14)	142 (0.11)	18 (0.13)	43 (0.14)
GH_44	1 (0.00)	0 (0.00)	0 (0.00)	0 (0.00)
GH_47	1 (0.00)	0 (0.00)	0 (0.00)	0 (0.00)
GH_48	2 (0.00)	0 (0.00)	16 (0.12)	8 (0.03)
GH_53	116 (0.10)	273 (0.22)	23 (0.17)	94 (0.31)
GH_57	2 (0.00)	5 (0.00)	0 (0.00)	0 (0.00)
GH_59	3 (0.00)	1 (0.00)	1 (0.01)	0 (0.00)
GH_63	0 (0.00)	1 (0.00)	0 (0.00)	0 (0.00)
GH_65	50 (0.04)	71 (0.06)	4 (0.03)	7 (0.02)
GH_66	3 (0.00)	1 (0.00)	0 (0.00)	1 (0.00)
GH_67	2 (0.00)	14 (0.01)	0 (0.00)	0 (0.00)
GH_70	2 (0.00)	0 (0.00)	0 (0.00)	0 (0.00)
GH_73	10 (0.01)	15 (0.01)	2 (0.01)	1 (0.00)
GH_77	144 (0.13)	133 (0.11)	6 (0.04)	34 (0.11)
GH_78	49 (0.04)	18 (0.01)	4 (0.03)	2 (0.01)
GH_81	0 (0.00)	12 (0.01)	0 (0.00)	0 (0.00)
GH_85	2 (0.00)	0 (0.00)	0 (0.00)	0 (0.00)
GH_88	68 (0.06)	51 (0.04)	14 (0.10)	19 (0.06)
GH_89	0 (0.00)	2 (0.00)	0 (0.00)	0 (0.00)
GH_92	0 (0.00)	1 (0.00)	0 (0.00)	0 (0.00)
GH_97	6 (0.01)	3 (0.00)	0 (0.00)	1 (0.00)
GH_98	2 (0.00)	2 (0.00)	0 (0.00)	0 (0.00)
GH_101	2 (0.00)	4 (0.00)	0 (0.00)	0 (0.00)
GH_106	1 (0.00)	2 (0.00)	0 (0.00)	1 (0.00)
GH_108	9 (0.01)	0 (0.00)	0 (0.00)	0 (0.00)
GH_115	6 (0.01)	4 (0.00)	0 (0.00)	0 (0.00)
GH_125	1 (0.00)	0 (0.00)	0 (0.00)	0 (0.00)
GH_127	64 (0.06)	59 (0.05)	12 (0.09)	19 (0.06)
GH_129	0 (0.00)	1 (0.00)	0 (0.00)	0 (0.00)
GH_130	8 (0.01)	12 (0.01)	1 (0.01)	0 (0.00)

Supplementary Table S5. Comparisons in relative abundances of COG functional categories among five metagenome groups: zoo carnivores' feces (ZC), zoo omnivores' feces (ZO), and zoo herbivores' feces (ZH), cow's rumen (CR), and flying squirrel's cecum (FS).

COG	functional category	ZC (%)	ZO (%)	ZH (%)	CR (%)	FS (%)	FDR
A	RNA processing and modification	0.01	0.01	0.00	0.02	0.00	0.229
B	Chromatin Structure and dynamics	0.02	0.02	0.02	0.02	0.01	0.374
C	Energy production and conversion	**6.56**	**6.06**	**6.48**	**7.09**	**4.78**	**0.040**
D	Cell cycle control	**1.28**	**1.37**	**1.45**	**1.26**	**1.64**	**0.043**
E	Amino acid transport and metabolism	**10.16**	**10.55**	**10.03**	**11.74**	**8.50**	**0.007**
F	Nucleotide transport and metabolism	**4.16**	**4.00**	**4.00**	**4.69**	**3.41**	**0.021**
G	Carbohydrate transport and metabolism	9.67	9.83	9.27	8.03	9.33	0.504
H	Coenzyme transport and metabolism	4.15	4.01	3.89	4.47	3.25	0.092
I	Lipid transport and metabolism	**2.42**	**2.42**	**2.85**	**3.15**	**2.72**	**0.001**
J	Translation	**8.00**	**8.93**	**9.71**	**12.37**	**10.45**	**0.000**
K	Transcription	**6.36**	**6.12**	**5.64**	**4.83**	**7.20**	**0.000**
L	Replication	7.83	8.39	8.70	8.62	9.79	0.065
M	Cell wall	**5.70**	**5.99**	**6.36**	**5.99**	**5.96**	**0.043**
N	Cell motility	0.85	0.52	0.49	0.50	0.61	0.878
O	Posttranslational modification	**3.51**	**3.63**	**3.82**	**4.80**	**3.55**	**0.003**
P	Inorganic ion transport and metabolism	**5.22**	**4.73**	**3.77**	**3.44**	**3.93**	**0.000**
Q	Secondary metabolites biosynthesis	**1.06**	**0.89**	**1.16**	**0.97**	**0.82**	**0.015**
R	General function prediction only	**9.78**	**9.99**	**10.30**	**8.34**	**9.98**	**0.003**
S	Function unknown	**4.97**	**4.79**	**4.34**	**3.32**	**4.66**	**0.002**
T	Signal transduction mechanisms	**3.89**	**3.30**	**3.18**	**2.80**	**3.70**	**0.004**
U	Intracellular trafficking	1.71	1.50	1.59	1.56	1.45	0.564
V	Defense mechanisms	**2.67**	**2.94**	**2.93**	**1.99**	**4.26**	**0.012**
W	Extracellular structures	0.01	0.00	0.00	0.00	0.00	0.374
Z	Cytoskeleton	**0.00**	**0.01**	**0.02**	**0.01**	**0.02**	**0.043**

Significant differences (FDR adjusted p-value < 0.05) revealed by Kruskal-Wallis test are shown in boldface.

Supplementary Table S6. Post-hoc pairwise comparisons in relative abundances of COG functional categories, focusing on the pairs between flying squirrel's cecum (FS) and other four metagenome groups: zoo carnivores' feces (ZC), zoo omnivores' feces (ZO), and zoo herbivores' feces (ZH), cow's rumen (CR).

COG	functional category	FDR	ZC vs. FS	ZO vs. FS	ZH vs. FS	CR vs. FS
A	RNA processing and modification	0.229				
B	Chromatin Structure and dynamics	0.374				
C	Energy production and conversion	**0.040**	0.064	0.134	**0.029**	0.061
D	Cell cycle control	**0.043**	**0.050**	0.068	0.171	0.078
E	Amino acid transport and metabolism	**0.007**	0.055	**0.014**	0.062	**0.003**
F	Nucleotide transport and metabolism	**0.021**	**0.033**	**0.049**	0.053	**0.004**
G	Carbohydrate transport and metabolism	0.504				
H	Coenzyme transport and metabolism	0.092				
I	Lipid transport and metabolism	**0.001**	0.115	0.118	0.441	0.395
J	Translation	**0.000**	**0.011**	**0.033**	0.180	0.262
K	Transcription	**0.000**	0.184	0.103	**0.005**	**0.001**
L	Replication	0.065				
M	Cell wall	**0.043**	0.436	0.596	0.318	0.491
N	Cell motility	0.878				
O	Posttranslational modification	**0.003**	0.417	0.431	0.157	**0.018**
P	Inorganic ion transport and metabolism	**0.000**	**0.039**	0.097	0.416	0.238
Q	Secondary metabolites biosynthesis	**0.015**	0.174	0.288	**0.045**	0.223
R	General function prediction only	**0.003**	0.275	0.461	0.257	0.089
S	Function unknown	**0.002**	0.400	0.448	0.192	**0.018**
T	Signal transduction mechanisms	**0.004**	0.406	0.143	0.084	**0.024**
U	Intracellular trafficking	0.564				
V	Defense mechanisms	**0.012**	**0.022**	**0.047**	**0.040**	**0.002**
W	Extracellular structures	0.374				
Z	Cytoskeleton	**0.043**	0.169	0.334	0.449	0.220

Dunn's test as a post-hoc test was conducted only for significant Kruskal-Wallis test (FDR < 0.05).

Significant pairwise comparisons ($p < 0.05$) are shown in boldface.

Supplementary Table S7. Comparisons in relative abundances of COG gene families associated with defense mechanisms among five metagenome groups: zoo carnivores' feces (ZC), zoo omnivores' feces (ZO), and zoo herbivores' feces (ZH), cow's rumen (CR), and flying squirrel's cecum (FS).

COG_ID	COG definition	ZC (%)	ZO (%)	ZH (%)	CR (%)	FS (%)	FDR
COG1132	ABC-type multidrug transport system	**0.763**	**0.812**	**0.711**	**0.524**	**1.801**	**0.043**
COG0534	Na+-driven multidrug efflux pump	**0.521**	**0.687**	**0.680**	**0.014**	**0.660**	**0.046**
COG1131	ABC-type multidrug transport system	0.305	0.257	0.271	0.281	0.589	0.236
COG1136	ABC-type antimicrobial peptide transport system	0.322	0.276	0.262	0.347	0.512	0.059
COG0286	Type I restriction-modification system	**0.071**	**0.115**	**0.137**	**0.087**	**0.165**	**0.043**
COG2274	ABC-type bacteriocin/lantibiotic exporters	**0.072**	**0.146**	**0.136**	**0.263**	**0.155**	**0.043**
COG0610	Type I site-specific restriction-modification system	**0.064**	**0.084**	**0.126**	**0.097**	**0.148**	**0.043**
COG0841	Cation/multidrug efflux pump	**0.294**	**0.272**	**0.344**	**0.192**	**0.088**	**0.046**
COG4096	Type I site-specific restriction-modification system	0.043	0.044	0.066	0.054	0.081	0.404
COG0577	ABC-type antimicrobial peptide transport system	**0.022**	**0.045**	**0.025**	**0.002**	**0.056**	**0.043**
COG0732	Restriction endonuclease S subunits	**0.005**	**0.018**	**0.016**	**0.003**	**0.055**	**0.043**
COG1968	Uncharacterized bacitracin resistance protein	0.060	0.060	0.052	0.036	0.052	0.282
COG1619	Homologs of microcin C7 resistance protein MccF	0.027	0.026	0.030	0.038	0.035	0.339
COG2720	Uncharacterized vancomycin resistance protein	0.015	0.029	0.043	0.035	0.031	0.059
COG1680	Beta-lactamase and other penicillin binding proteins	**0.043**	**0.046**	**0.079**	**0.075**	**0.030**	**0.043**
COG1566	Multidrug resistance efflux pump	**0.090**	**0.063**	**0.059**	**0.009**	**0.021**	**0.043**
COG1401	GTPase subunit of restriction endonuclease	**0.003**	**0.006**	**0.010**	**0.000**	**0.019**	**0.043**
COG3587	Restriction endonuclease	0.028	0.032	0.028	0.016	0.019	0.591
COG4823	Abortive infection bacteriophage resistance protein	0.012	0.017	0.013	0.004	0.018	0.355
COG1787	Endonuclease related to Mrr restriction enzymes	**0.001**	**0.002**	**0.006**	**0.000**	**0.013**	**0.043**
COG2367	Beta-lactamase class A	0.009	0.017	0.006	0.001	0.012	0.075
COG4268	McrBC 5-methylcytosine restriction system	**0.002**	**0.007**	**0.010**	**0.001**	**0.010**	**0.043**
COG4767	Glycopeptide antibiotics resistance protein	**0.014**	**0.008**	**0.006**	**0.000**	**0.009**	**0.043**
COG2348	Uncharacterized protein for methicillin resistance	0.012	0.050	0.010	0.008	0.007	0.236
COG3023	Negative regulator of beta-lactamase expression	0.018	0.011	0.007	0.021	0.006	0.457
COG1715	Restriction endonuclease	0.004	0.006	0.005	0.004	0.006	0.959
COG3183	Predicted restriction endonuclease	0.002	0.003	0.001	0.002	0.005	0.287
COG4403	Lantibiotic modifying enzyme	0.005	0.003	0.001	0.000	0.004	0.199
COG4845	Chloramphenicol O-acetyltransferase	0.006	0.009	0.012	0.003	0.004	0.457
COG1403	Restriction endonuclease	0.006	0.005	0.003	0.002	0.003	0.597
COG2602	Beta-lactamase class D	**0.010**	**0.002**	**0.001**	**0.000**	**0.002**	**0.043**

COG3896	Chloramphenicol 3-O-phosphotransferase	0.000	0.000	0.001	0.000	0.002	0.199
COG1002	Type II restriction enzyme, methylase subunits	**0.000**	**0.001**	**0.000**	**0.000**	**0.001**	**0.046**
COG2746	Aminoglycoside N3'-acetyltransferase	0.004	0.002	0.008	0.012	0.001	0.059
COG3570	Streptomycin 6-kinase	**0.002**	**0.000**	**0.000**	**0.002**	**0.000**	**0.043**
COG4171	ABC-type antimicrobial peptide transport system	0.008	0.001	0.000	0.001	0.000	0.061
COG2810	Predicted type IV restriction endonuclease	0.000	0.000	0.000	0.000	0.000	0.076
COG0842	ABC-type multidrug transport system	0.023	0.010	0.003	0.000	0.000	0.185
COG3725	Membrane protein for beta-lactamase induction	0.005	0.001	0.000	0.000	0.000	0.198
COG4170	ABC-type antimicrobial peptide transport system	0.002	0.001	0.000	0.000	0.000	0.199
COG4168	ABC-type antimicrobial peptide transport system	0.004	0.001	0.000	0.000	0.000	0.199
COG3440	Predicted restriction endonuclease	0.004	0.003	0.001	0.000	0.000	0.287
COG4257	Streptogramin lyase	0.000	0.000	0.000	0.000	0.000	0.404
COG4167	ABC-type antimicrobial peptide transport system	0.002	0.001	0.000	0.002	0.000	0.570
COG4452	Membrane protein involved in colicin E2 resistance	0.005	0.003	0.004	0.006	0.000	0.850

Significant differences (FDR adjusted p-value < 0.05) revealed by Kruskal-Wallis test are shown in boldface.

Supplementary Table S8. Comparisons in relative abundances of glycoside hydrolase (GH) groups among five metagenome groups: zoo carnivores' feces (ZC), zoo omnivores' feces (ZO), and zoo herbivores' feces (ZH), cow's rumen (CR), and flying squirrel's cecum (FS).

	ZC (%)	ZO (%)	ZH (%)	CR (%)	FS (%)	FDR
GH_1	**0.232**	**0.265**	**0.059**	**0.070**	**0.171**	**0.025**
GH_2	**0.236**	**0.262**	**0.348**	**0.203**	**0.224**	**0.024**
GH_3	**0.183**	**0.325**	**0.345**	**0.293**	**0.422**	**0.024**
GH_4	0.055	0.026	0.045	0.045	0.024	0.132
GH_5	**0.004**	**0.041**	**0.063**	**0.021**	**0.059**	**0.013**
GH_6	0.005	0.003	0.002	0.000	0.002	0.257
GH_8	0.012	0.004	0.007	0.002	0.002	0.556
GH_9	0.011	0.018	0.028	0.026	0.013	0.197
GH_10	**0.028**	**0.063**	**0.085**	**0.053**	**0.019**	**0.024**
GH_11	0.000	0.001	0.004	0.006	0.000	0.059
GH_13	0.233	0.288	0.211	0.251	0.312	0.093
GH_15	**0.001**	**0.000**	**0.000**	**0.000**	**0.002**	**0.013**
GH_16	**0.003**	**0.031**	**0.025**	**0.005**	**0.008**	**0.014**
GH_18	0.020	0.013	0.013	0.014	0.020	0.552
GH_19	0.000	0.000	0.000	0.000	0.000	0.743
GH_20	0.090	0.085	0.115	0.079	0.005	0.119
GH_24	**0.007**	**0.005**	**0.001**	**0.000**	**0.003**	**0.047**
GH_25	**0.034**	**0.063**	**0.040**	**0.011**	**0.064**	**0.024**
GH_26	**0.001**	**0.013**	**0.030**	**0.010**	**0.011**	**0.013**
GH_27	0.050	0.113	0.074	0.141	0.052	0.059
GH_28	0.008	0.032	0.030	0.008	0.036	0.052
GH_29	0.067	0.064	0.095	0.094	0.017	0.296
GH_30	0.009	0.016	0.013	0.008	0.023	0.507
GH_31	0.098	0.120	0.138	0.117	0.064	0.303
GH_32	0.083	0.091	0.049	0.059	0.014	0.110
GH_33	0.000	0.000	0.000	0.000	0.000	0.336
GH_35	0.021	0.021	0.024	0.028	0.016	0.874
GH_36	**0.074**	**0.145**	**0.122**	**0.225**	**0.103**	**0.046**
GH_37	0.012	0.008	0.004	0.000	0.001	0.414
GH_38	0.047	0.037	0.052	0.034	0.014	0.154
GH_39	0.000	0.011	0.011	0.012	0.015	0.093
GH_42	0.031	0.053	0.054	0.041	0.071	0.286

GH_43	**0.075**	**0.106**	**0.128**	**0.024**	**0.129**	**0.025**
GH_44	0.000	0.000	0.002	0.000	0.000	0.119
GH_45	0.000	0.000	0.001	0.000	0.000	0.277
GH_46	0.000	0.001	0.000	0.000	0.000	0.620
GH_47	**0.001**	**0.000**	**0.000**	**0.000**	**0.000**	**0.046**
GH_48	0.000	0.001	0.004	0.001	0.001	0.295
GH_52	0.000	0.000	0.000	0.000	0.000	0.620
GH_53	**0.013**	**0.040**	**0.039**	**0.055**	**0.161**	**0.024**
GH_57	0.004	0.011	0.017	0.005	0.003	0.257
GH_59	**0.000**	**0.000**	**0.001**	**0.000**	**0.002**	**0.024**
GH_63	0.000	0.001	0.001	0.000	0.000	0.815
GH_64	0.000	0.001	0.000	0.000	0.000	0.889
GH_65	0.090	0.050	0.059	0.048	0.051	0.735
GH_66	0.001	0.014	0.005	0.003	0.002	0.336
GH_67	0.009	0.018	0.019	0.075	0.006	0.093
GH_68	0.001	0.000	0.000	0.000	0.000	0.876
GH_70	0.004	0.018	0.000	0.000	0.001	0.186
GH_73	**0.041**	**0.043**	**0.020**	**0.007**	**0.010**	**0.024**
GH_75	0.000	0.000	0.001	0.000	0.000	0.735
GH_76	0.001	0.002	0.002	0.001	0.000	0.889
GH_77	0.098	0.140	0.137	0.158	0.117	0.493
GH_78	**0.003**	**0.020**	**0.023**	**0.021**	**0.029**	**0.041**
GH_81	0.001	0.000	0.000	0.000	0.005	0.121
GH_85	0.006	0.005	0.002	0.000	0.001	0.093
GH_88	0.034	0.046	0.066	0.019	0.051	0.132
GH_89	0.022	0.016	0.023	0.027	0.001	0.146
GH_92	**0.045**	**0.085**	**0.118**	**0.129**	**0.000**	**0.042**
GH_97	**0.022**	**0.061**	**0.080**	**0.083**	**0.004**	**0.024**
GH_98	0.002	0.005	0.002	0.000	0.002	0.414
GH_101	**0.024**	**0.002**	**0.001**	**0.000**	**0.002**	**0.041**
GH_102	0.003	0.000	0.000	0.000	0.000	0.414
GH_106	**0.007**	**0.030**	**0.060**	**0.031**	**0.001**	**0.024**
GH_108	0.001	0.000	0.002	0.002	0.004	0.667
GH_114	0.000	0.001	0.000	0.000	0.000	0.276
GH_115	**0.003**	**0.030**	**0.038**	**0.041**	**0.004**	**0.013**
GH_116	**0.000**	**0.000**	**0.005**	**0.002**	**0.000**	**0.024**
GH_125	0.019	0.019	0.017	0.043	0.000	0.119

GH_127	**0.043**	**0.078**	**0.119**	**0.092**	**0.052**	**0.024**
GH_129	0.002	0.000	0.000	0.000	0.000	0.493
GH_130	**0.004**	**0.011**	**0.020**	**0.010**	**0.008**	**0.043**

Significant differences (FDR adjusted p-value < 0.05) revealed by Kruskal-Wallis test are shown in boldface.
