# Supplementary material for: Functional Characteristics of the Flying Squirrel's Cecal Microbiota under a Leaf-Based Diet, Based on Multiple Meta-Omic Profiling
Source: Front Microbiol. 2018 Jan 4;8:2622. doi: 10.3389/fmicb.2017.02622 (PMC5758534; doi:10.3389/fmicb.2017.02622)

Supplementary Figures

Functional characteristics of the flying squirrel's cecal microbiota under a leaf-based diet, based on multiple meta-omic profiling

Hsiao-Pei Lu[1,#], Po-Yu Liu[1,2], Yu-bin Wang[1,3], Ji-Fan Hsieh[1,§], Han-Chen Ho[4], Shiao-Wei Huang[1], Chun-Yen Lin[3], Chih-hao Hsieh[1,5,6.7], and Hon-Tsen Yu[1,2,*]

[1]Department of Life Science, National Taiwan University, Taipei, Taiwan

[2]Genome and Systems Biology Degree Program, National Taiwan University & Academia Sinica, Taipei, Taiwan

[3]Institute of Information Science, Academia Sinica, Taipei, Taiwan

[4]Department of Anatomy, Tzu Chi University, Hualien, Taiwan

[5]Institute of Oceanography, National Taiwan University, Taipei, Taiwan

[6]Institute of Ecology and Evolutionary Biology, National Taiwan University, Taipei, Taiwan

[7]National Center for Theoretical Sciences, Taipei, Taiwan

[#]Present address: Institute of Oceanography, National Taiwan University, Taipei, Taiwan

[§]Present address: Division of Ecology and Evolution, Research School of Biology, The Australian National University, Canberra, Australia

[*]Correspondence:

Hon-Tsen Yu

E-mail: ayu@ntu.edu.tw

Supplementary Figure S1. Sampling locations from the proximal to distal ends of the flying squirrel’s digestive tract.

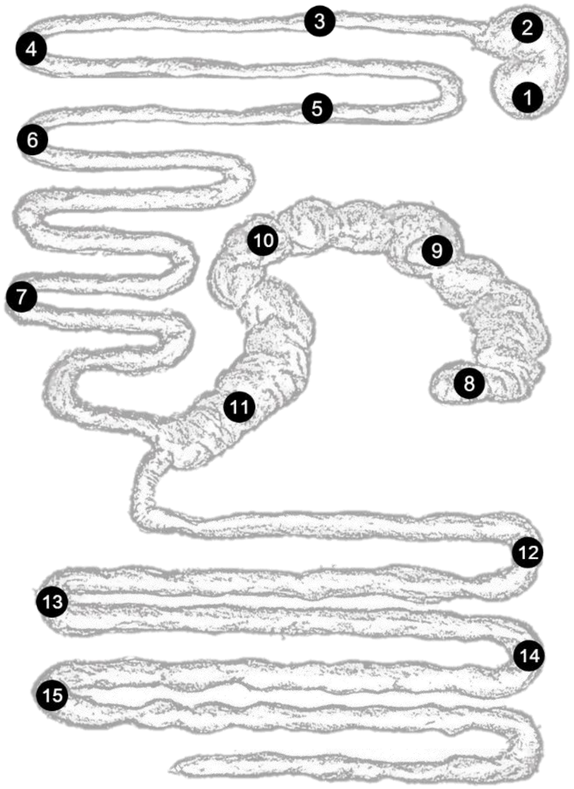

Supplementary Figure S2. Distribution of feed particle sizes in four main gut compartments: the stomach (a), small intestine (b), cecum (c), and large intestine (d) of the flying squirrel. Red lines are the fitting curves based on a normal distribution.

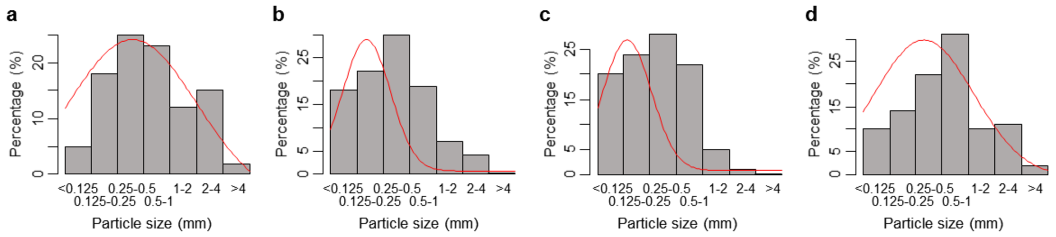

Supplementary Figure S3. Texture of feed contents from the stomach (a) and cecum (b) of the flying squirrel.

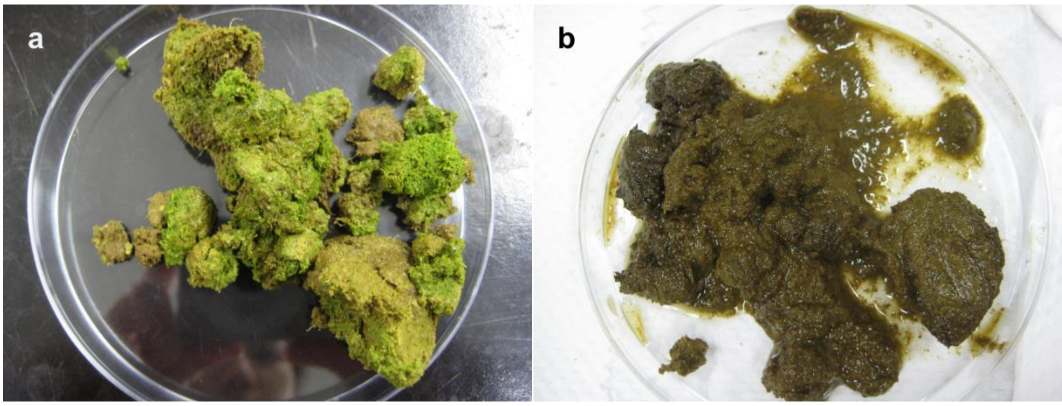

Supplementary Figure S4. Mean relative intensity of detected phytochemical compounds across gut regions of the flying squirrel, for the whole group of phytochemicals (a) or each subgroup (b-f). Signal intensity of each compound was normalized between 0 to 1, according to its relative proportion in the four gut compartments. Mean relative intensity was the average of signal intensities for compounds detected in a sample; the error bars represent the standard error of the mean (SEM).

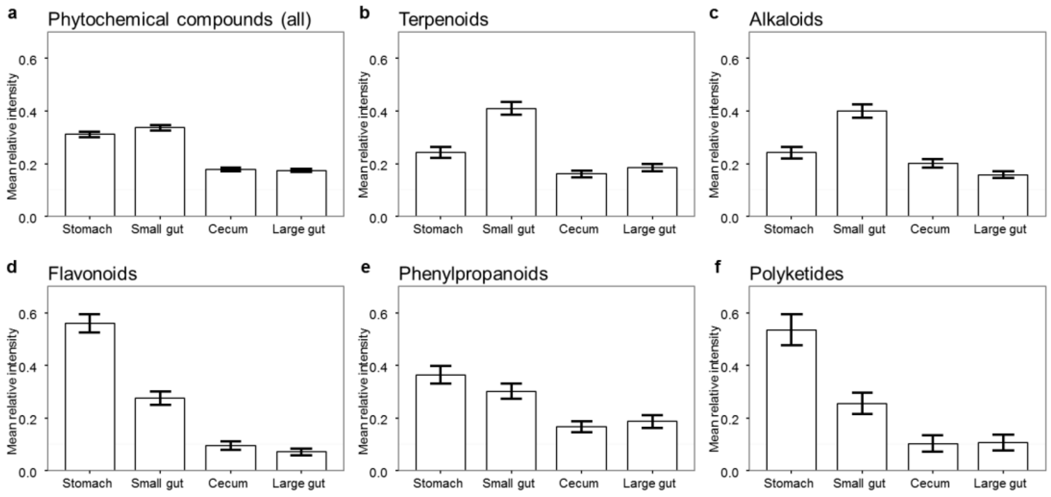

Supplementary Figure S5. Functional profiles based on COG functional categories for metagenomes (DNA-level) and metatranscriptomes (RNA-level) of the cecal microbiota from two flying squirrels (FS1 and FS2). Among these categories, J, K, L are associated with information storage and processing; D, M, N, O, T, U, V are associated with cellular processes and signaling; C, E, F, G, H, I, P, Q are associated with metabolism; R and S are poorly characterized.

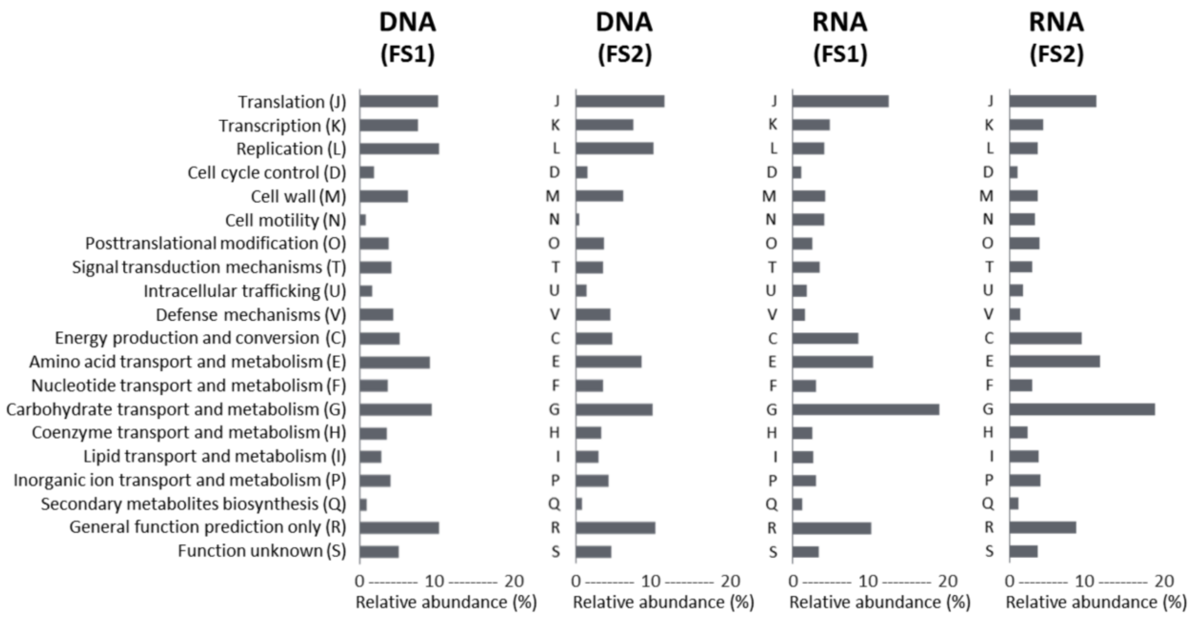

Supplementary Figure S6. Sequences mapped to KEGG pathways for metagenomes (DNA-level) and metatranscriptomes (RNA-level) of the cecal microbiota from two flying squirrels (FS1 and FS2). Only pathways containing more than 10 KOs and having > 50% coverage (detected KOs / total KOs in the pathway) in either one library are shown.

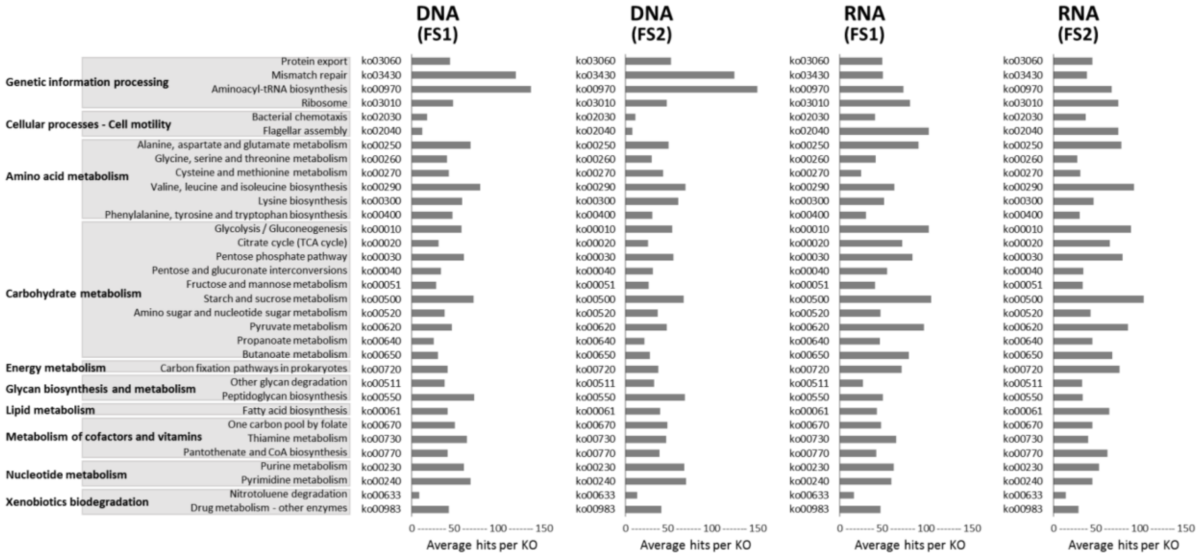

Supplementary Figure S7. The KEGG map for bacterial chemotaxis, showing detected genes and compounds in the metagenome, metatranscriptome, and metabolome of the flying squirrel's cecal microbiota. Colors: genes identified in both the metagenome and metatranscriptome are in yellow; genes only identified in the metagenome are in pink; and compounds identified in the metabolome are in red.

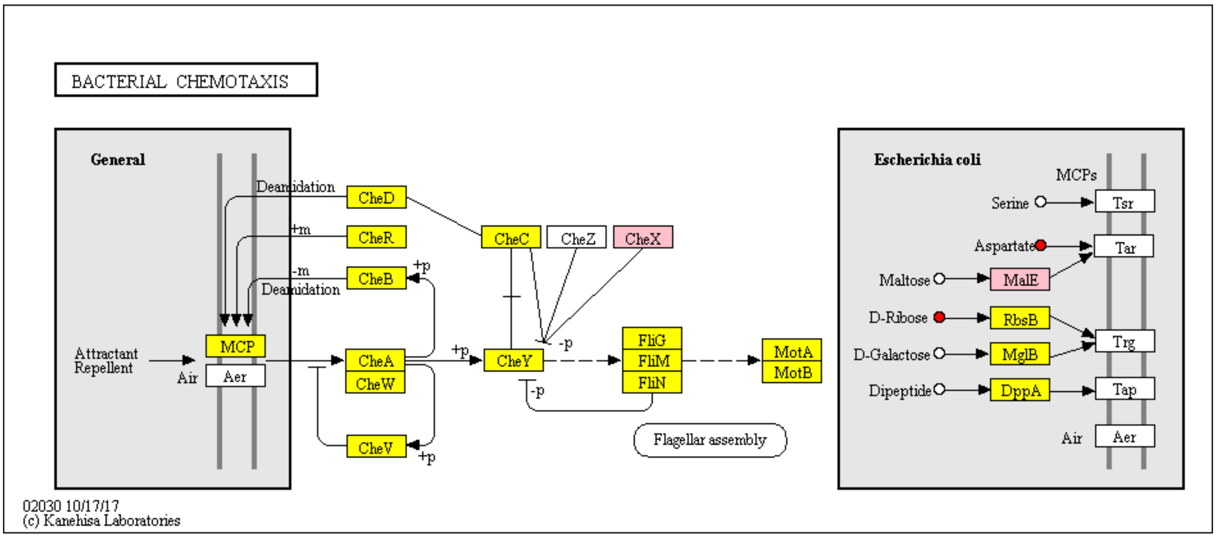

Supplementary Figure S8. The KEGG map for flagellar assembly, showing detected gene components in both the metagenome and metatranscriptome of the flying squirrel's cecal microbiota (marked in yellow). Notably, the three undetected components (i.e. FlgF, FlgH and FlgI) were only required for gram-negative bacteria. According to taxonomic annotation, the flying squirrel's cecal microbiota were predominated by gram-positive *Firmicutes* taxa.

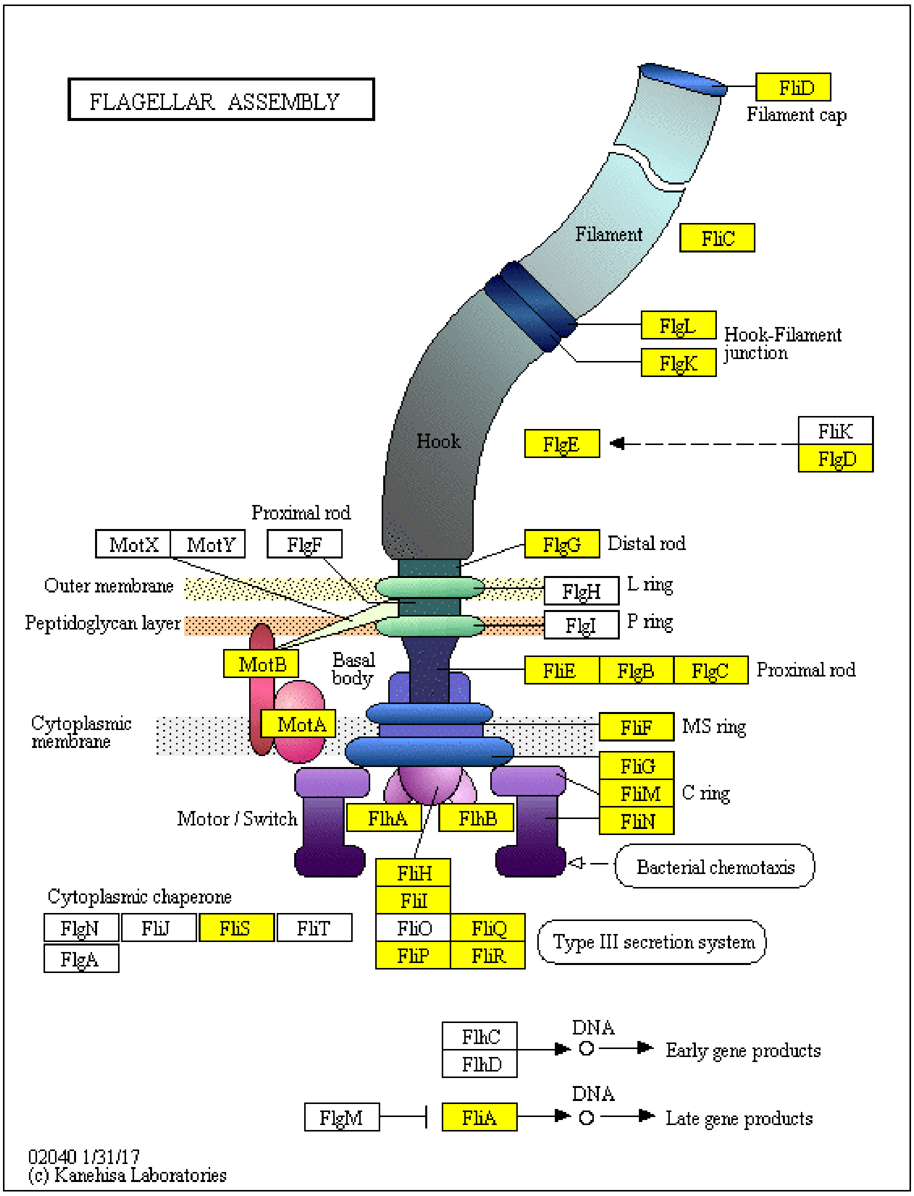

Supplementary Figure S9. The KEGG map for ABC transporters associated with sugar import, showing detected genes and compounds in the metagenome, metatranscriptome, and metabolome of the flying squirrel's cecal microbiota. Colors: genes identified in both the metagenome and metatranscriptome are in yellow; genes only identified in the metagenome are in pink; genes only identified in the metatranscriptome are in green; and compounds identified in the metabolome are in red.

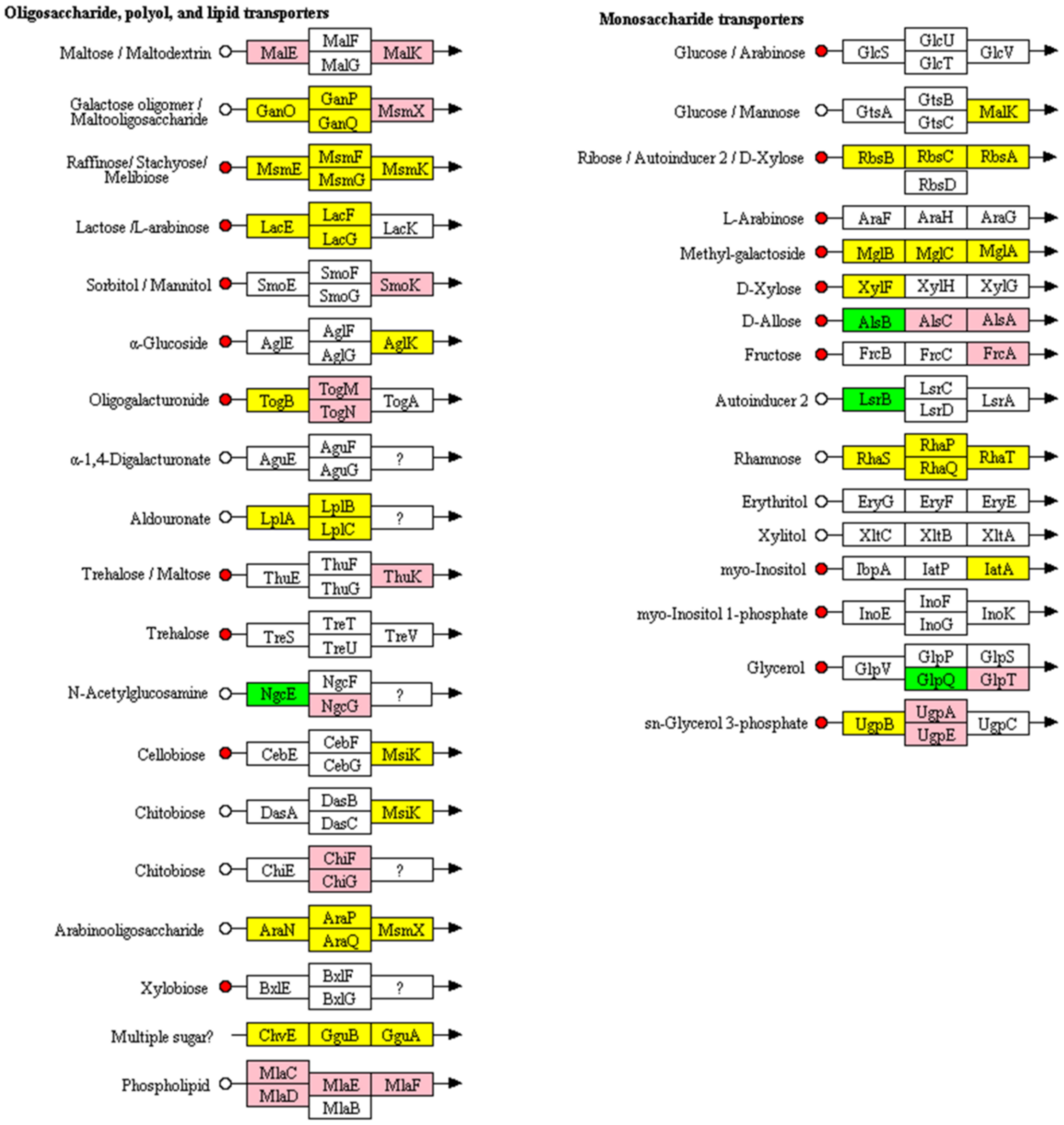

Supplementary Figure S10. The KEGG map for pentose and glucuronate interconversions, showing detected genes and compounds in the metagenome, metatranscriptome, and metabolome of the flying squirrel's cecal microbiota. Colors: genes identified in both the metagenome and metatranscriptome are in yellow; genes only identified in the metagenome are in pink; and compounds identified in the metabolome are in red.

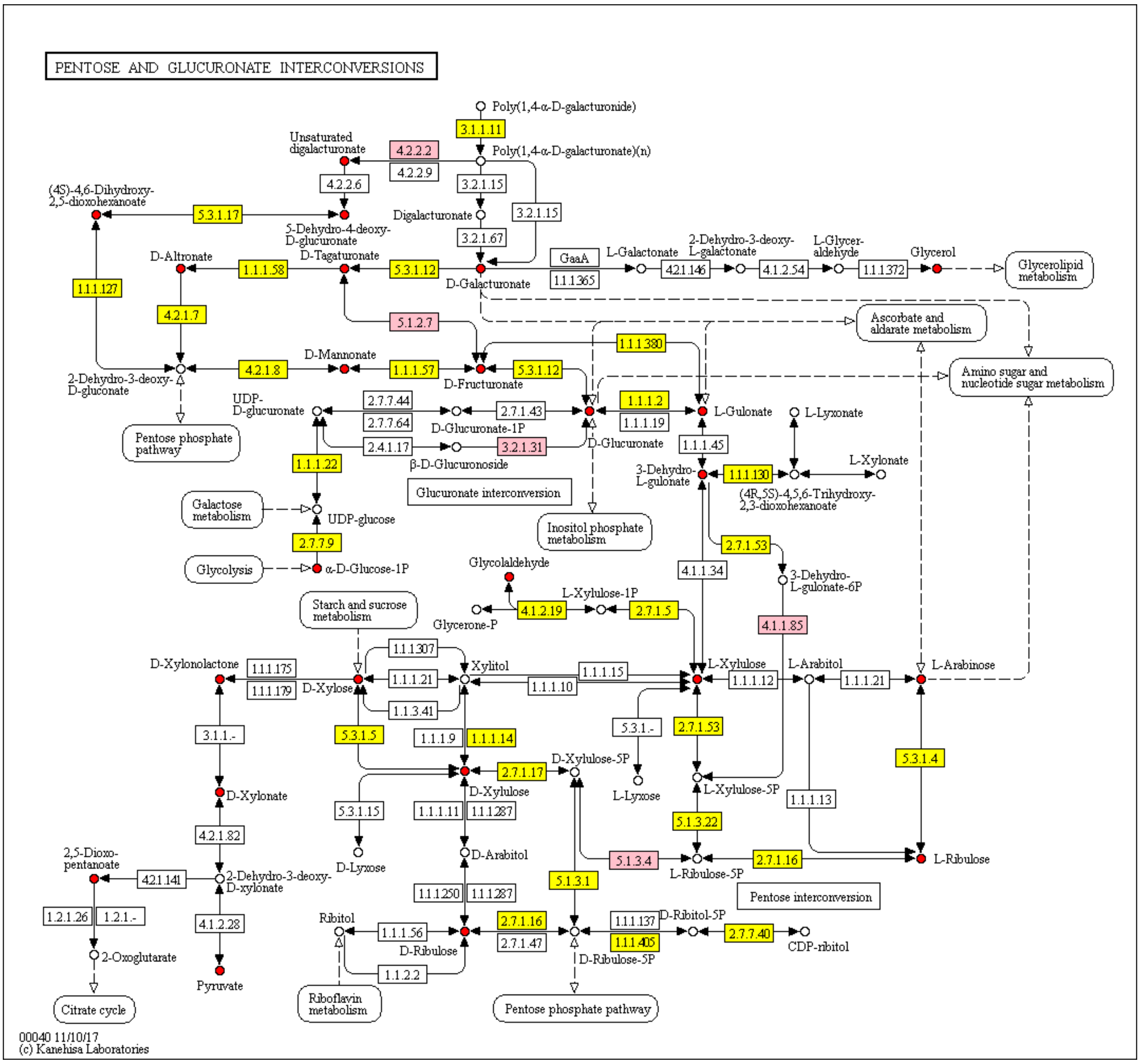

Supplementary Figure S11. The KEGG map for pentose phosphate pathway, showing detected genes and compounds in the metagenome, metatranscriptome, and metabolome of the flying squirrel's cecal microbiota. Colors: genes identified in both the metagenome and metatranscriptome are in yellow; genes only identified in the metagenome are in pink; genes only identified in the metatranscriptome are in green; and compounds identified in the metabolome are in red.

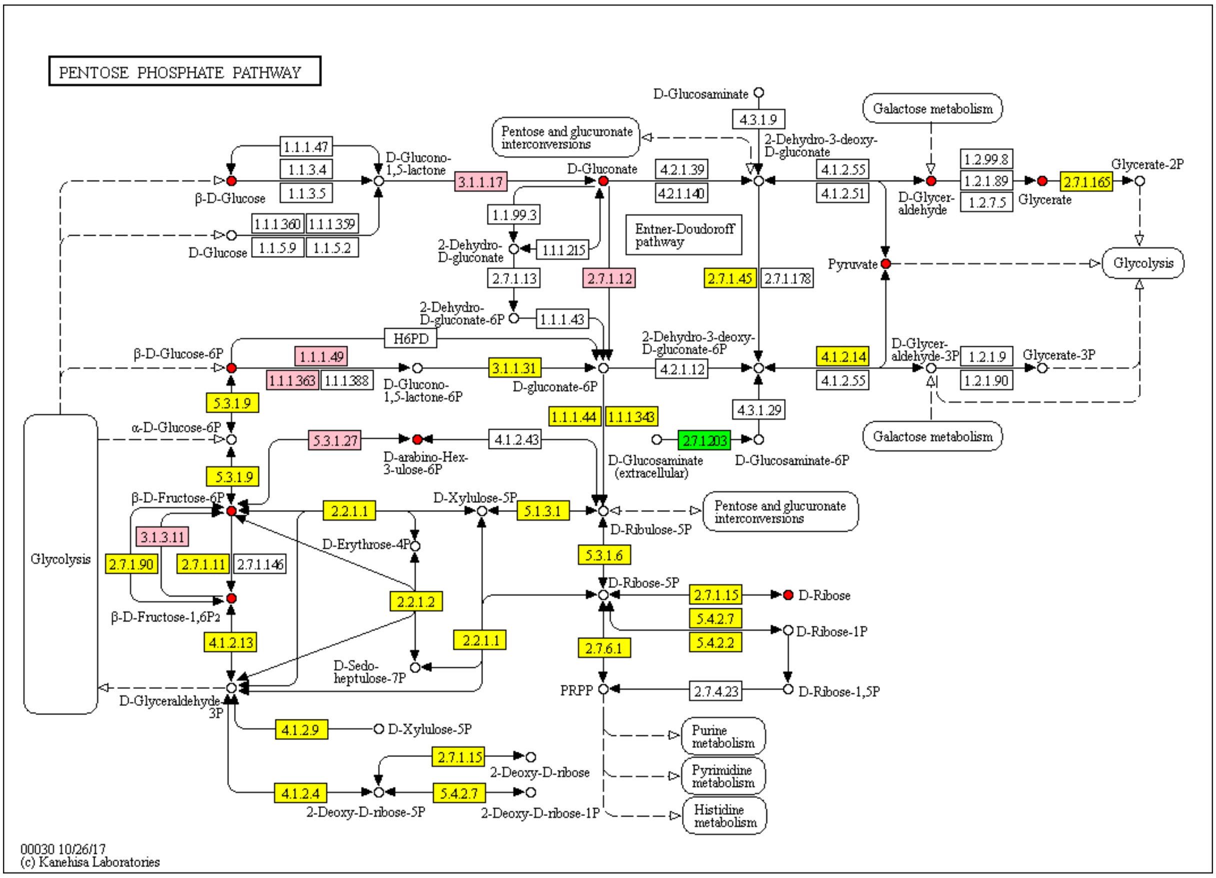

Supplementary Figure S12. The KEGG map for glycolysis (EMP pathway), showing detected genes and compounds in the metagenome, metatranscriptome, and metabolome of the flying squirrel's cecal microbiota. Colors: genes identified in both the metagenome and metatranscriptome are in yellow; genes only identified in the metagenome are in pink; genes only identified in the metatranscriptome are in green; and compounds identified in the metabolome are in red.

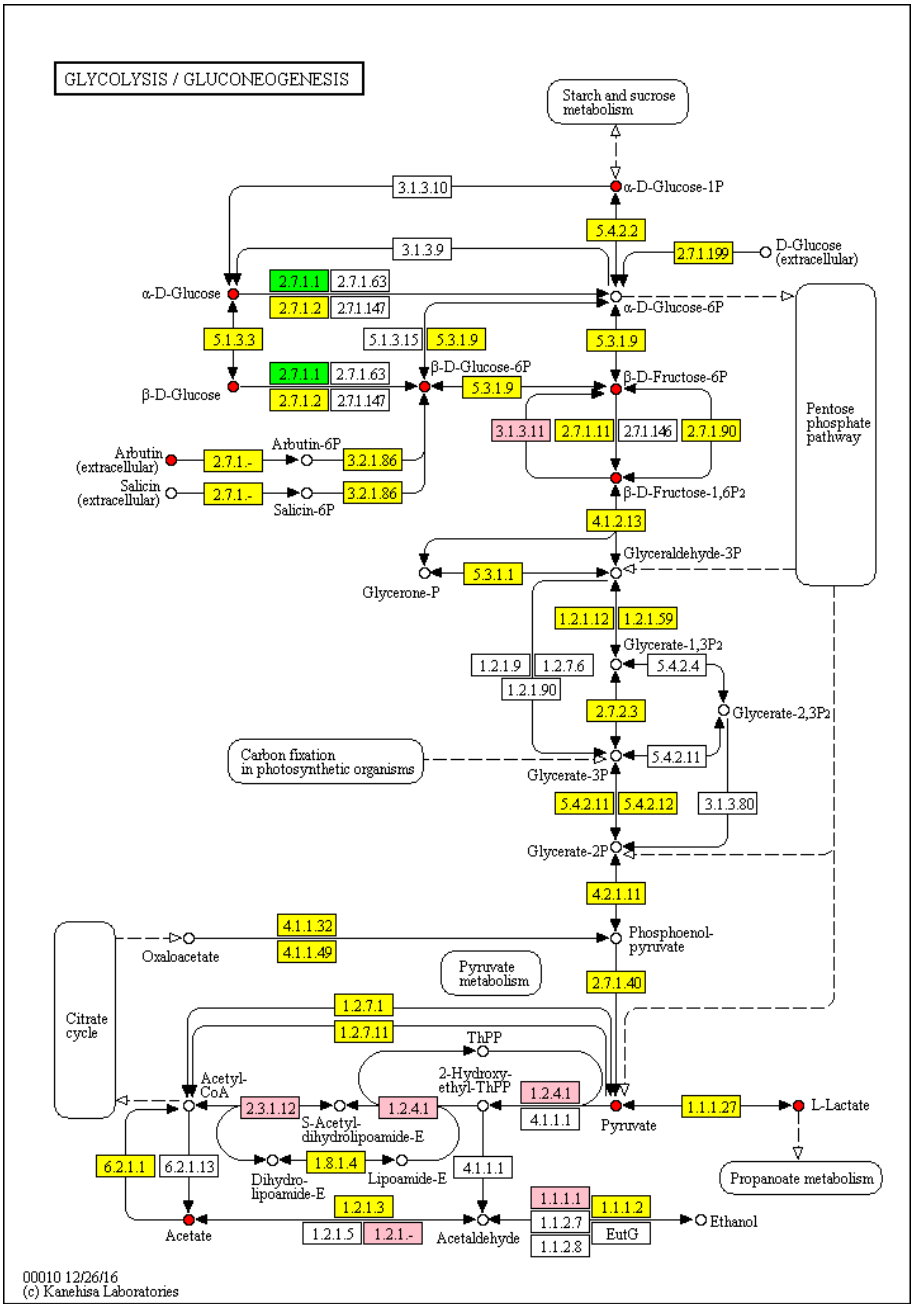

Supplementary Figure S13. The KEGG map for biosynthesis of amino acids, showing detected genes and compounds in the metagenome, metatranscriptome, and metabolome of the flying squirrel's cecal microbiota. Colors: genes identified in both the metagenome and metatranscriptome are in yellow; genes only identified in the metagenome are in pink; genes only identified in the metatranscriptome are in green; and compounds identified in the metabolome are in red.

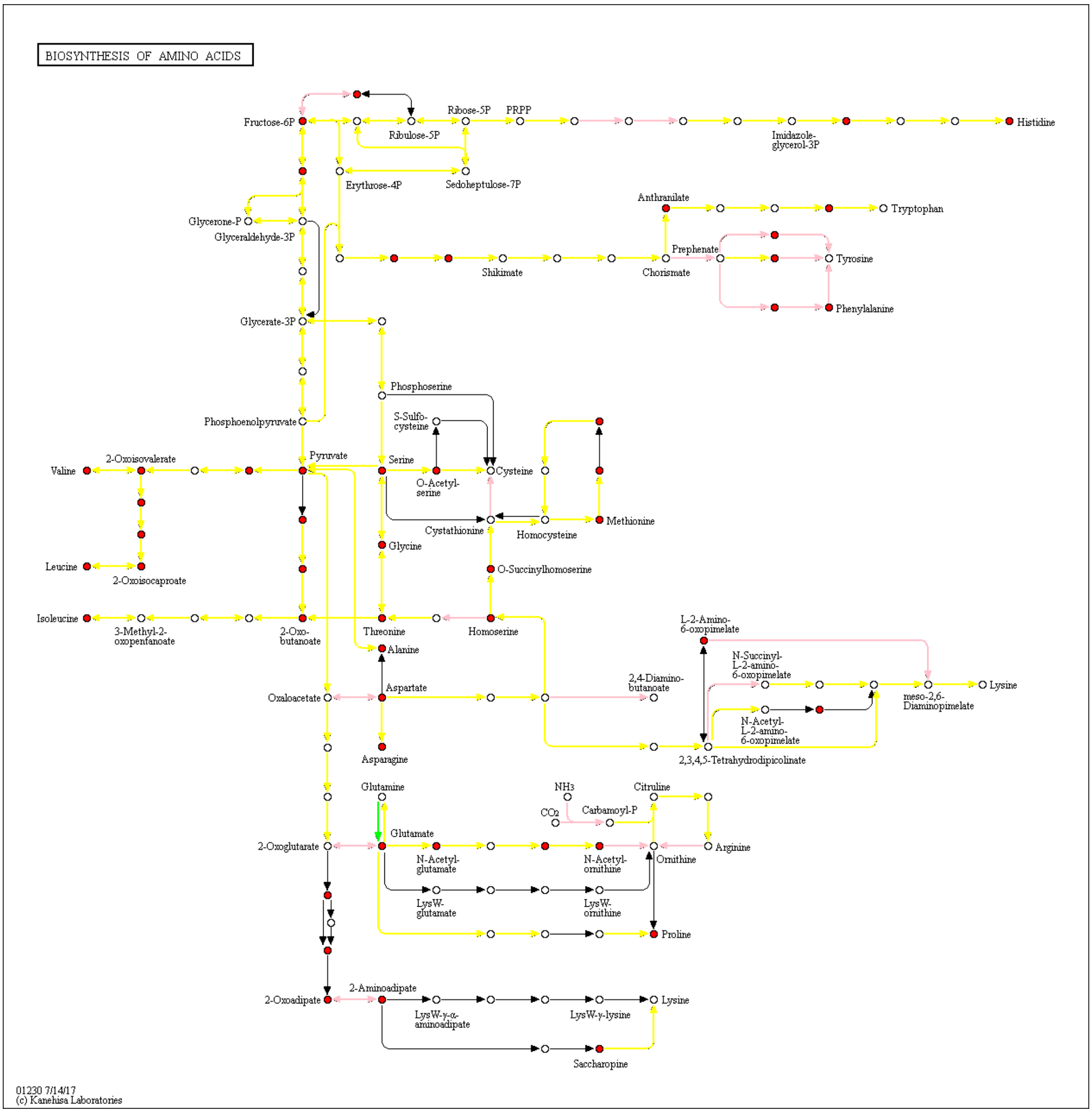

Supplementary Figure S14. The KEGG map for purine metabolism, showing detected genes and compounds in the metagenome, metatranscriptome, and metabolome of the flying squirrel's cecal microbiota. Colors: genes identified in both the metagenome and metatranscriptome are in yellow; genes only identified in the metagenome are in pink; genes only identified in the metatranscriptome are in green; and compounds identified in the metabolome are in red.

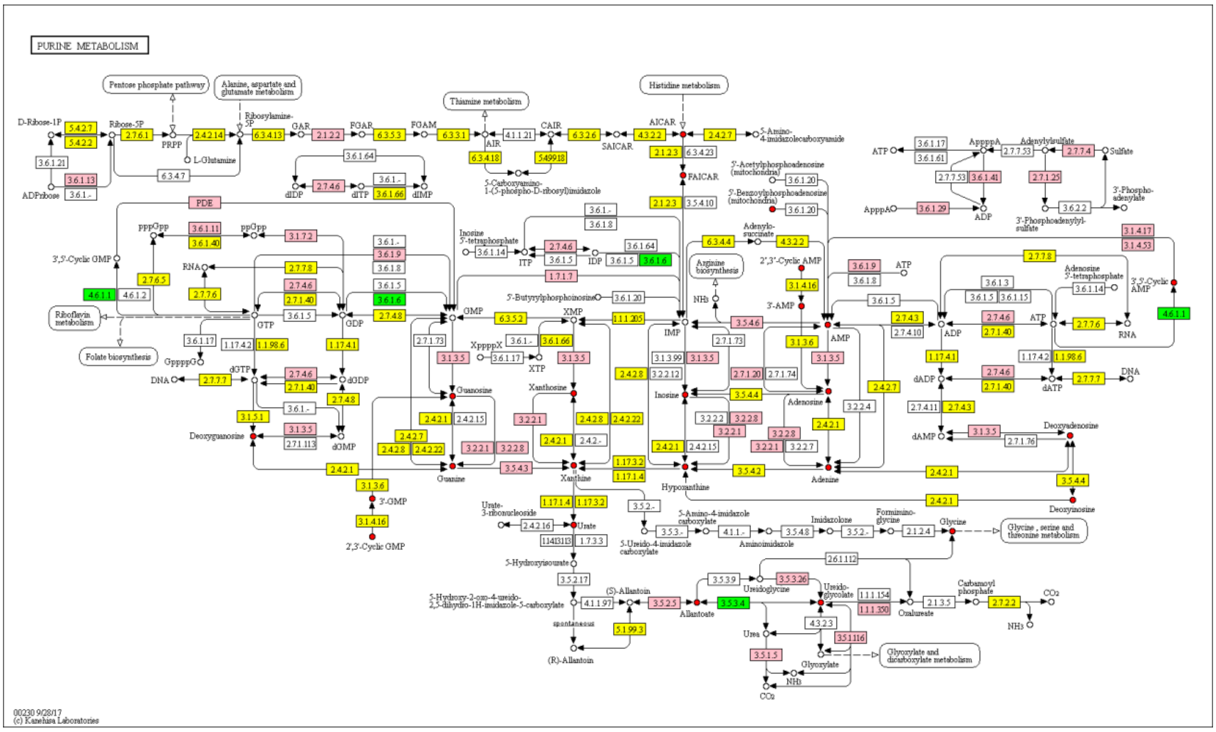

Supplementary Figure S15. The KEGG map for pyrimidine metabolism, showing detected genes and compounds in the metagenome, metatranscriptome, and metabolome of the flying squirrel's cecal microbiota. Colors: genes identified in both the metagenome and metatranscriptome are in yellow; genes only identified in the metagenome are in pink; genes only identified in the metatranscriptome are in green; and compounds identified in the metabolome are in red.

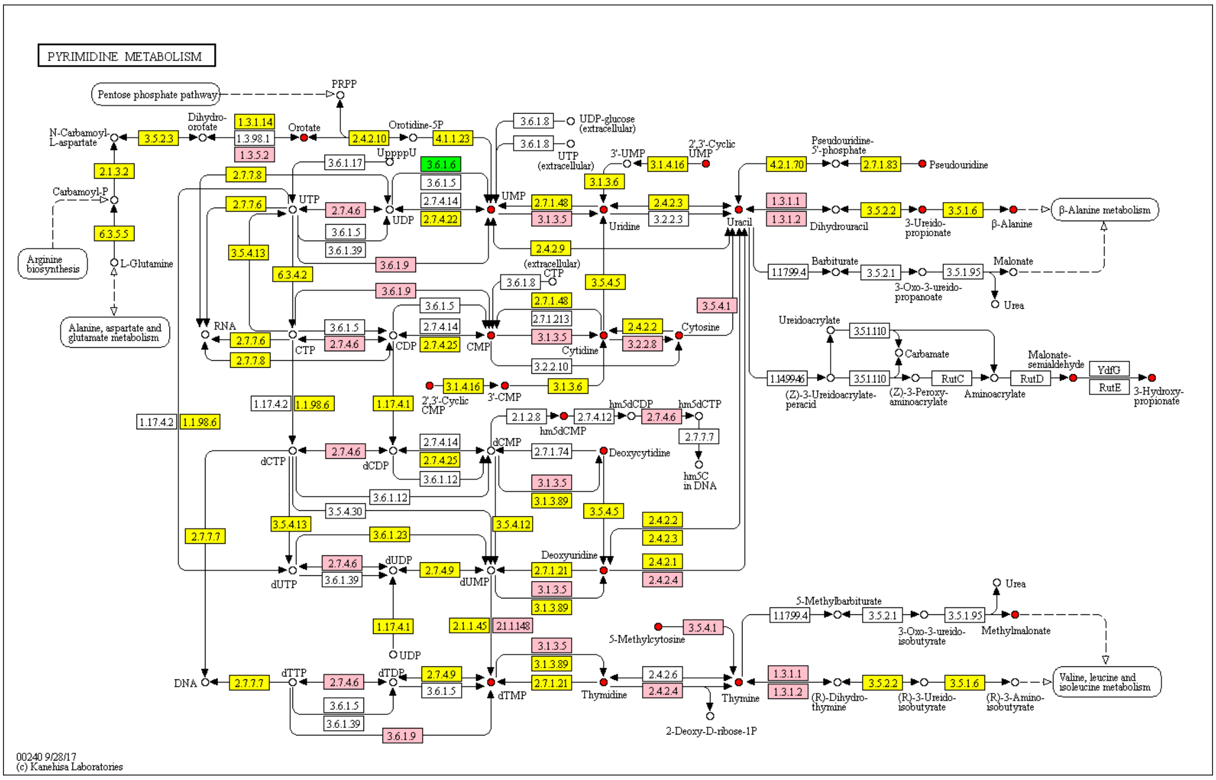

Supplementary Figure S16. The KEGG map for peptidoglycan biosynthesis, showing detected genes and compounds in the metagenome, metatranscriptome, and metabolome of the flying squirrel's cecal microbiota. Colors: genes identified in both the metagenome and metatranscriptome are in yellow; genes only identified in the metagenome are in pink; and compounds identified in the metabolome are in red.

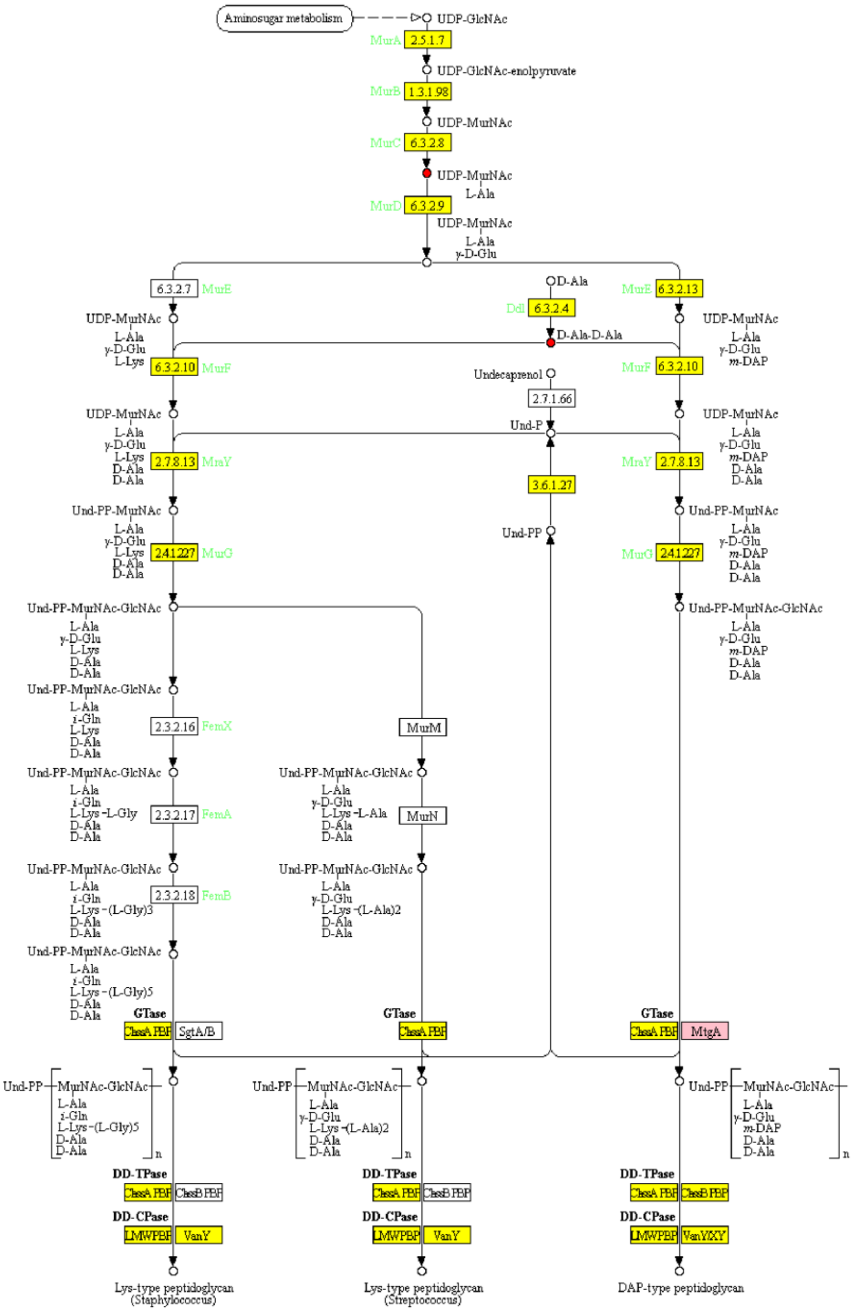

Supplementary Figure S17. NMDS plot based on Pfam compositions, revealing functional similarity / dissimilarity among mammalian gut metagenomes. In addition to 2 datasets from the flying squirrel (FS) cecum of this study, publicly available gut metagenomes, including 4 datasets from the cow rumen and 39 datasets from the fecal samples of zoo mammals (categorized by their diets: carnivore, omnivore, and herbivore), were included for the comparison.

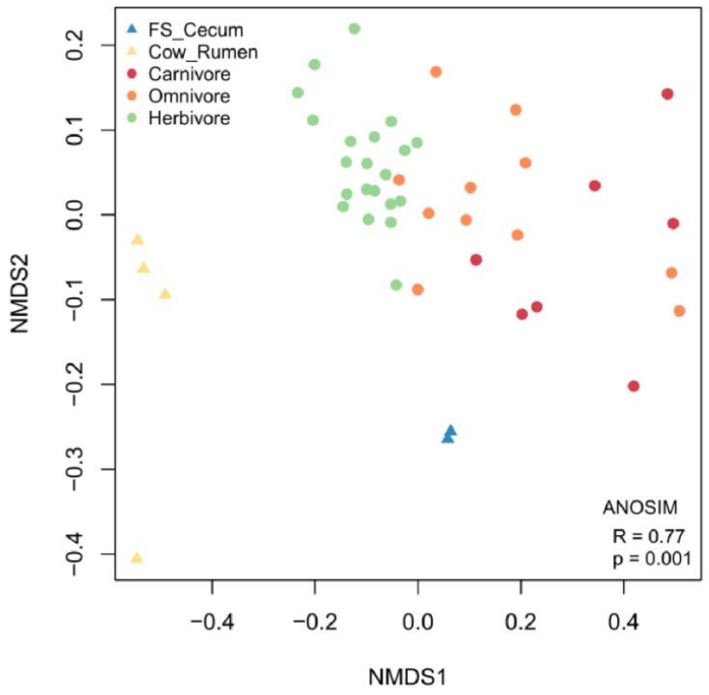

Supplement: Supplementary file 2 [file Image1.pdf]
